# Co-production practice and future research priorities in United Kingdom-funded applied health research: a scoping review

**DOI:** 10.1186/s12961-022-00838-x

**Published:** 2022-04-02

**Authors:** Helen Smith, Luke Budworth, Chloe Grindey, Isabel Hague, Natalie Hamer, Roman Kislov, Peter van der Graaf, Joe Langley

**Affiliations:** 1 NIHR Applied Research Collaboration Yorkshire and Humber, Bradford, United Kingdom; 2 Bradford Institute for Health Research, Bradford, United Kingdom; 3 Faculty of Medical Sciences, Newcastle University, Newcastle, United Kingdom; 4 Faculty of Business and Law Manchester, Metropolitan University, Manchester, United Kingdom; 5 School of Health Sciences, The University of Manchester, Manchester, United Kingdom; 6 NIHR Applied Research Collaboration Greater Manchester, Manchester, United Kingdom; 7 NIHR Applied Research Collaboration North East and North Cumbria, Cumbria, United Kingdom; 8 School of Health and Life Sciences, Teeside University, Middlesbrough, United Kingdom; 9 Lab4Living, Sheffield Hallam University, Sheffield, United Kingdom

**Keywords:** Co-production, Co-creation, Applied health research, Scoping review

## Abstract

**Background:**

Interest in and use of co-production in healthcare services and research is growing. Previous reviews have summarized co-production approaches in use, collated outcomes and effects of co-production, and focused on replicability and reporting, but none have critically reflected on how co-production in applied health research might be evolving and the implications of this for future research. We conducted this scoping review to systematically map recent literature on co-production in applied health research in the United Kingdom to inform co-production practice and guide future methodological research.

**Methods:**

This scoping review was performed using established methods. We created an evidence map to show the extent and nature of the literature on co-production and applied health research, based on which we described the characteristics of the articles and scope of the literature and summarized conceptualizations of co-production and how it was implemented. We extracted implications for co-production practice or future research and conducted a content analysis of this information to identify lessons for the practice of co-production and themes for future methodological research.

**Results:**

Nineteen articles reporting co-produced complex interventions and 64 reporting co-production in applied health research met the inclusion criteria. Lessons for the practice of co-production and requirements for co-production to become more embedded in organizational structures included (1) the capacity to implement co-produced interventions, (2) the skill set needed for co-production, (3) multiple levels of engagement and negotiation, and (4) funding and institutional arrangements for meaningful co-production. Themes for future research on co-production included (1) who to involve in co-production and how, (2) evaluating outcomes of co-production, (3) the language and practice of co-production, (4) documenting costs and challenges, and (5) vital components or best practice for co-production.

**Conclusion:**

Researchers are operationalizing co-production in various ways, often without the necessary financial and organizational support required and the right conditions for success. We argue for accepting the diversity in approaches to co-production, call on researchers to be clearer in their reporting of these approaches, and make suggestions for what researchers should record. To support co-production of research, changes to entrenched academic and scientific practices are needed.

Protocol registration details: The protocol for the scoping review was registered with protocols.io on 19 October 2021: https://dx.doi.org/10.17504/protocols.io.by7epzje.

**Supplementary Information:**

The online version contains supplementary material available at 10.1186/s12961-022-00838-x.

## Background

Despite the lack of clarity around the definition, what it means in practice and what it comprises, enthusiasm for co-production in healthcare services and research is growing. The lack of clarity is evident in the plethora of terms in use. For example, within healthcare we witness services, programmes and interventions being “co-created”, “co-designed”, “co-evaluated” or “co-implemented”. This can involve stakeholder and public engagement through participation or involvement in any or all steps of the applied research cycle [1, 2]. All are regarded as processes of co-production, but the way they are enacted and operationalized varies depending on the purpose, what is being co-produced and by whom [3, 4]. Some of the ambiguity in co-production also comes from its unclear relationship with patient and public involvement/and engagement (PPI/E). For some, co-production represents enhanced PPI/E, a way to improve on its shortcomings by re-engaging with the principles of power-sharing, equality and social justice, and reinforcing the democratic right of citizens to influence healthcare [3, 5]. For others, co-production simply represents another way of consulting the public and service users to provide instrumental inputs into health and social care services and research, demonstrating a more technocratic rationale [6]. New experimental perspectives on co-production, which frame it as a generative process and a social space within which new interactions, insights and knowledge are produced, challenge conventional notions of engagement and involvement [4]. However, whilst new conceptualizations and discussion can help the approach and foundational principles to further develop and evolve, and more and different forms of co-production to emerge, this also adds to the uncertainty around its use.

The United Kingdom National Institute for Health Research (NIHR) recently embraced co-production as a means of improving public involvement in research, framing it as a more collaborative and egalitarian mode of involvement with values and principles for greater equality [7]. Unlike other funders of health research globally, NIHR insists on community involvement in research proposals, and it is a key criterion for funding [8]. Other funders have started to encourage co-production by providing flexible funding to cover costs of user-led research design and engagement [9] and funding research into best practice for community engagement [10]. In the United Kingdom context, some argue that the architecture of the new NIHR Applied Research Collaboration funding model enables authentic and visible co-production [11]. Others are more cautious, arguing that co-production can only be as successful as the system allows, and that traditional research structures often fail to facilitate effective public involvement, leading to co-opting of the term co-production without making a tangible difference [12, 13]. However, there are anecdotal stories of successful collaborative working from the previous NIHR funding model, Collaborations for Leadership in Applied Health Research and Care (CLAHRC), where co-production projects added value and led to the implementation of novel services and interventions [14]. Success stories like these are not always published or reported on or described in a way that explicates how best to support researchers to co-produce applied health research or complex health interventions.

Recent systematic reviews of co-production have summarized the different co-production approaches in use and collated outcomes and effects of co-production, and some have focused specifically on replicability and reporting. Slattery et al. conducted a rapid overview of reviews, specifically of research co-design (defined as involvement of research users at the study planning phase only) and its effectiveness, and found that co-design is widely used but rarely reported or evaluated in detail [15]. Another review examining the use of experience-based co-design (EBCD) in health service improvement also found inconsistent reporting and variation in the use of the approach, leading the authors to argue for reporting guidelines to encourage consistency and to improve the potential of the approach [13]. Halvorsrud pooled effects data from co-creation projects in international health research and found moderate to small effects on a range of outcomes from different study designs and interventions, yet little evidence of longer-term effects of co-creation [16]. Acknowledging the lack of evidence of the impact of co-produced or co-created interventions in healthcare settings, some authors have reviewed the evidence on outcomes and factors influencing the quality and level of co-production and co-creation [17, 18]. These reviews found that studies of processes and factors influencing co-production dominated, and identified fewer studies evaluating clinical, service or cost outcomes.

While various aspects of co-production have been subject to more or less rigorous systematic reviews in the last 5 years, no reviews have targeted co-produced applied health research or the co-production of complex interventions (which is often the focus of applied research). Nor have previous reviews critically reflected on how co-production is conceptualized in applied health research, or how the principles are enacted, to draw out implications for the practice of co-production and for future research. Applied health research is becoming more collaborative, with patient and public groups increasingly engaged in research projects alongside academics and practitioners, and funders are gradually mandating the use of co-production principles. It is therefore timely to reflect on what has been learned about the practice of co-production in applied health research and help forecast the direction of future research.

We conducted a scoping review to systematically map recent literature on co-production in applied health research in the United Kingdom to inform co-production practice and guide future methodological research. The review was designed to answer the following questions:

1. What is the type and scope of literature on co-production in applied health research?
2. How is co-production conceptualized and understood?
3. How is co-production implemented in applied health research?
4. What lessons are there for co-production practice and future research, based on the current knowledge base?

## Methods

We used established scoping review methods to systematically map the nature of the evidence, summarize practice, and identify gaps in the literature on co-production in applied health research [19, 20]. We had to streamline our approach to the study screening and selection process because of time and resource availability, and therefore followed accepted rapid review methods for single screening of titles and abstracts and independent verification of a sample of full-text articles [20]. We intentionally kept the review questions broad and open to generate breadth of coverage, and once we had a sense of the volume of literature, we set parameters to limit the number of studies to a manageable level. The protocol is published on protocols.io.

We define co-production as a way for academics, practitioners, and patients and the public to work together, sharing power and responsibility across the whole research cycle [7]. For the purpose of this scoping review, we have assumed that co-production happens at any or all stages of the research cycle, and so included reports using any of the plethora of terms in use including co-design, co-production, co-implementation, co-evaluation and co-creation.

### Search strategy

We followed a standard approach to locate published literature in scoping reviews [21]. First, we listed key terms and synonyms relevant to each of the inclusion criteria (Table 1) and performed an initial high-level search of one relevant multidisciplinary database (ProQuest) using main keywords in the title. We analysed the text words used in the retrieved article titles and abstracts, then conducted a comprehensive search of five other relevant databases (CINAHL, Google Scholar, MEDLINE, Scopus, Web of Science) using all identified keywords and index terms. We conducted a separate search to ensure we identified co-production of complex health interventions as well as the broader applied health research literature. The third step involved searching all reference lists of retrieved articles to identify additional literature. An example search strategy can be found in Additional file 1. We downloaded all retrieved articles and managed the screening process in Mendeley.

**Table 1:** Scoping review inclusion criteria

Inclusion criteria	Definition	Synonyms and search terms
Participants	Any stakeholders involved in applied health research (e.g. researchers, patients, public)	Health research, applied health research^a^, health, healthcare, health care, complex health intervention research^b^
Intervention	Co-production approach or methodology	Co-production, co-produc*, co-design, co-creation, co-creat*, co-evaluation, co-evaluat*
Context	United Kingdom literature: research conducted in or relevant to United Kingdom context (e.g. systematic reviews that included studies conducted in the United Kingdom)	Limit = United Kingdom
Outcomes	Definitions, typologies or conceptualization of co-production Key outcomes (conceptual, methodological, impact, health, experiential) Research implications	
Type of literature	Any type of published literature including systematic reviews, literature reviews, empirical research (evaluations of co-production or co-produced intervention research), guidelines, opinion or comment pieces	
Language	English language only	Limit = English language
Date limits	From 2010 onwards, when “co-production” started to appear in the health literature	Limit to year = “2010–2020” Subsequently limited to 2018–2020 given the large number of hits from initial searches

^a^Applied health research aims to address the immediate issues facing the health and social care system, bringing research evidence into practice and influencing policy

^b^Interventions with multiple behavioural, technological and organizational interacting components and nonlinear causal pathways and components that act independently or interdependently

### Study selection

We included any type of published literature (empirical research, reviews, guidelines, opinion pieces or commentaries) relevant to co-production in applied health research or complex intervention development that reported on a range of outcomes including conceptual, methodological, impact or health. We were interested in literature that included definitions or conceptualizations of co-production, as well as implications for future research. We intentionally included only papers reporting applied health research conducted in the United Kingdom—to keep the focus on learning within a specific context. Following the initial searches and familiarity with the extent of the literature, we refined our inclusion criteria. Our initial database searches included papers published from 2010 onwards, when “co-production” began to appear in the health literature and as a requirement of some funding schemes in the United Kingdom; we subsequently limited the date range to 2018–2020 due to the large number of hits and to keep the charting and summarizing steps manageable.

Based on established rapid review methods [20], one author (HS) applied the inclusion criteria to all titles and abstracts retrieved in the search. After excluding articles that did not meet the criteria, we retrieved full text copies of all remaining articles. One author screened these for inclusion (HS), and another author (LB) independently screened 25% of articles; discrepancies in include or exclude decisions were resolved by discussion.

### Data extraction

We used a Microsoft Excel worksheet to chart the characteristics and record key information from the articles included in the review (e.g. author, year of publication, study design, health speciality, aim, intervention type, outcomes reported, implications for practice and research). The items and information to be collected from each article were piloted by two team members, and adjustments made to ensure it was fit for purpose and standard information could be extracted in the same way for each article. Charting was completed by three authors (CG, IH, AH) and an independent check of 25% of the articles was done by another author (HS).

### Summarizing and reporting the findings

We used a descriptive-analytical method using the charted information as an overall framework for reporting across all included articles [19]. The resulting chart or evidence map shows the extent and nature of the literature on co-production and applied health research. Based on this map we developed a narrative summary, first describing the characteristics of the articles and scope of the literature (type, study design, health speciality, key outcomes reported), followed by a summary of conceptualizations of co-production and how co-production was implemented, as described in the articles. We extracted from the discussion section of each study any mention of implications for co-production practice or future research and conducted a content analysis of this information to identify lessons for the practice of co-production and themes for future methodological research. Reporting of the findings follows the Preferred Reporting Items for Systematic reviews and Meta-Analyses extension for Scoping Reviews (PRISMA-ScR) format [22].

## Findings

### Description of included studies

**Fig. 1.**
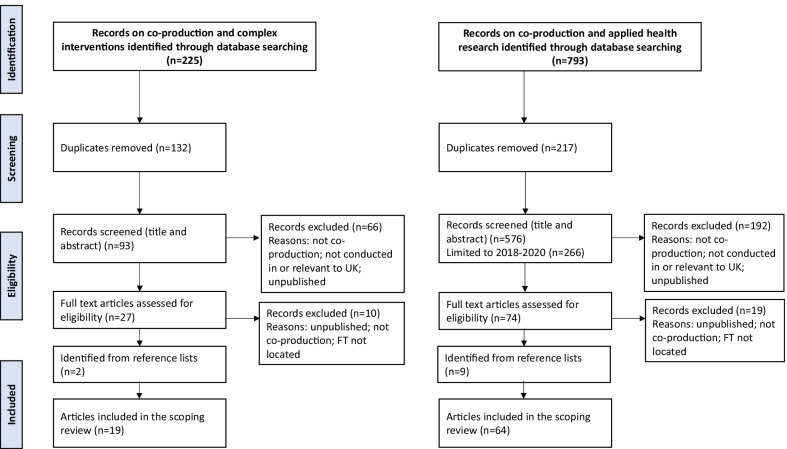
Adapted Preferred Reporting Items for Systematic Reviews and Meta-Analyses flow diagram of the search strategy

Database searching identified 793 records on co-production and applied health research and 225 on co-produced complex interventions (after limiting the search to 2018–2020). After removal of duplicates, there were 576 records on co-production and applied health research and 93 on complex interventions, of which we reviewed the full texts of 74 and 27, respectively. We excluded articles if they did not report on co-production, were not conducted in or relevant to the United Kingdom context, or were unpublished reports (Fig. 1). After including additional relevant articles identified from reference lists, *n* = 19 articles reporting co-produced complex interventions and *n* = 64 reporting co-production in applied health research met the inclusion criteria and were included in the scoping review.

### Scope of literature on co-production in applied health research

Table 2 summarizes the key characteristics of studies included in the scoping review. Nineteen reported co-produced complex interventions (*N* = 19) including intervention development or evaluation studies (*n* = 10), systematic reviews or evidence reviews (*n* = 3) and critical reflections or opinions (*n* = 6). The intervention studies mainly used descriptive study designs, including mixed-method observational studies that described the development of co-produced interventions or qualitative research that reported the process of co-producing an intervention and/or stakeholder views on the process. The systematic reviews or rapid evidence reviews synthesized empirical evaluations, processes and outcomes of co-production, and the critical reflections or opinion pieces described author experiences of co-producing interventions, or provided interpretations and conceptualizations of co-production.

**Table 2:** Characteristics of included studies

Author (year)	Lead organization/location	Aim	Study design/stakeholder type	Intervention reported	Health specialty	Outcomes of interest	Type of co-production used/methods	Co-production features/principles^a^	Reports research or methodology gaps	Reports policy/practice implications	Funder or source of funding
(i) Co-produced complex interventions (*N* = 19)											
Intervention development or evaluation (*n* = 10)											
Brookes [23]	NHS Trusts Birmingham Nottingham	To develop a reflective learning framework and toolkit for healthcare staff to improve patient, family and staff experience	Observational/mixed-method Clinical and managerial staff, patients and relatives from acute medical units	Patient experience and reflective learning (PEARL) toolkit—locally adaptable workplace-based toolkit with guidance on using reflective learning to incorporate patient and staff experience in routine clinical activities	Acute and intensive care	Impact Barriers and facilitators of reflective behaviours Observations of capability, opportunity & motivation of staff Output reflective learning toolkit	Co-design Meetings and workshops with all participants Reflection and discussion	Sharing of power Joint decision-making Involvement at all project stages		*	NIHR
Buckley 2019 [24]	University NW England	To explore the preliminary effects and acceptability of a co-produced physical activity referral intervention	Evaluation	Physical activity referral intervention designed to support participants in making gradual and sustainable changes to their physical activity levels	Public health/health promotion	Health Physical activity, cardiometabolic and anthropometric measures Impact Perception of the intervention vs usual care	Co-design	NR	*		PhD studentship
Buckley 2018 [25]	University NW England	To report process data from the participatory co-development phase of an exercise referral scheme (ERS) in a large city in NW England	Qualitative/participatory research Multilevel: commissioners, general practitioners (GPs), health trainers, exercise referral practitioners, academics	Physical activity referral intervention designed to support participants in making gradual and sustainable changes to their physical activity levels	Public health/health promotion	Impact Challenges of co-production Output Factors to consider when translating evidence into practice in an exercise referral setting	Service co-production Development group meetings Small group collaborative activities	Sharing of power Respecting & valuing all contributions Ongoing dialogue Continuous reflection	*	*	PhD studentship
Clayson 2018 [26]	Community research organization Liverpool	To create a working aide-mémoire, using accessible language, for the process of co-production research between academia and marginalized and stigmatized groups (e.g. people with lived experience of substance use recovery)	Qualitative/ethnographic reflection Academic and community researchers	Checklist to guide co-production	Addiction/substance use	Methodological Problems and factors to ensure adherence to co-production principles	Co-production Video diaries Blogs Recorded interviews Critical reflection	Knowledge exchange Asset-sharing Respecting & valuing all contributions Joint decision-making Continuous reflection Involvement at all project stages	*	*	NR
Davies 2019 [27]	University London	To report the development and components of a prototype website to support family caregivers of a person with dementia towards the end of life	Observational/mixed-method Academics, health workers, carers, charity members with expertise in dementia	Prototype website aimed at supporting family caregivers of someone with dementia towards the end of life in the United Kingdom	Older people/dementia	Output Targets and components of the website	Co-production Research development group meetings User testing in individual interviews	Involvement at all project stages Including all perspectives		*	NIHR
Evans 2019 [25]	University Swansea	To report the method used by a group of patient and carer service users to develop and implement a model for involving public members in research	Observational/mixed-method Patients with chronic long-term condition and carers	Service Users with Chronic Conditions Encouraging Sensible Solutions (SUCCESS) model for co-production that involves service users from the start	Chronic illness	Methodological Process of co-production Output Principles for involving service users	Co-production One workshop with group work	Including all perspectives Establishing ground rules Involving public members in research	*	*	NIHR
Farr 2018 [28]	University Bristol	To examine patient and staff views, experiences and acceptability of a United Kingdom primary care online consultation system	Evaluation/mixed-method GPs, practice nurses, practice managers, administrators, patients	eConsult online consultation system for primary care	Primary care	Impact Patient interaction with and use of eConsult; staff satisfaction; practice efficiency Health Consultation type and outcome	Service co-production Used as a theoretical framework for analysis of interviews	NR	*	*	NIHR
Gradinger 2019 [29]	University NHS Trust Devon	To report on the impact of two researchers in residence (RiR) working on care model innovations in an integrated care provider organization, as perceived by stakeholders	Case study/mixed-method RiR, academics, quality improvement lead, managers, clinicians	Two new care models: (1) Enhanced Intermediate Care Service and (2) co-located holistic link-worker Wellbeing Coordinators Programme	Social care	Impact Stakeholder perceptions of impact; attributes and behaviours for effective interaction	Co-production using embedded researchers Collaborative working	Ongoing dialogue Building and maintaining relationships	*	*	Torbay Medical Research Fund Torbay & an NHS Foundation Trust supported by NIHR
Henshall 2018 [30]	University West Midlands	To improve quality and content of midwives’ discussions with low-risk women on place of birth	Observational/mixed-method Academics, midwives, women’s representatives	Place of birth intervention package	Maternal health	Impact Midwives’ use and impact of package; knowledge and confidence in providing information to women	Co-design Feedback visits to midwives (led by academics) Workshops with midwives and women’s reps (separately then together)	Including all perspectives	*	*	NIHR
Hubbard 2020 [31]	University Scottish Highlands	To quickly develop an intervention to support people with severe mental ill health, that is systematic, and based on theory and evidence	Observational/mixed-method Academics, health practitioners, charity representatives	“Nature Walks for Wellbeing” Recently discharged mental health patients are supported to go on nature walks to support their long-term recovery	Mental health	Output Nature Walks for Wellbeing, a 60-min walk in a group Booklet outlining the importance of outdoor activity Text message once/week for the first 12 weeks post-discharge to support patients	Co-production Meetings between academics and stakeholders	Including all perspectives Joint decision-making Respecting & valuing all contributions	*	*	Supported by NIHR
Systematic or evidence reviews or overviews (*n* = 3)											
Lim 2020 [32]	University Global	To describe the process and outcomes of services or products co-produced with patients in hospital settings	Rapid evidence review	NA	Health services research	Impact Co-production strategies and types Outcomes associated with co-produced interventions Methodological limitations within the co-production process	Co-production (various)	NA	*	*	National Health and Medical Research Council Fellowship
O’Cathain 2019 [33]	University England	To review approaches to intervention development to identify the range of approaches available in order to help researchers to develop complex interventions	Systematic methods overview	NA	Health services research	Output Creation of a taxonomy/guide for intervention development approaches	Partnership approaches (incl co-production, co-creation, EBCD)	NA	*		Medical Research Council
Smith 2018 [34]	University United Kingdom	To produce an updated synthesis of the co-creation and co-production evidence base in the United Kingdom by identifying empirical evaluations of policies, programmes, interventions and services which incorporated principles of co-creation and co-production	Rapid evidence review	NA	Health services research	Methodological Definitions, objectives and methods used to evaluate co-created and co-produced policies, programmes and interventions	Co-production Co-creation	NA	*		NR
Critical reflections or opinion (*n* = 6)											
Locock 2019 [35]	University England	To examine the boundaries and commonalities between co-design approaches to incorporating user perspectives (in the context of designing biomedical research interventions)	Opinion	NA	Biomedical research	Conceptual Identifying overlap between methods/concepts Ethical/conceptual underpinnings	Co-production Co-design	NA	*	*	National Science Foundation
Madden 2020 [36]	University York	To explore how PPI and co-production were interpreted and applied in the development of a complex intervention on alcohol and medicine use in community pharmacies	Critical reflection Pharmacists, patients, carers, PPI group, professional practice group, policy advisory group	Community pharmacy: Highlighting Alcohol use in Medication appointments (CHAMP)-1 programme	Pharmacy	Methodological Barriers/levers to co-producing an intervention in a NIHR research programme	Co-production Workshops with pharmacists and patients Consultation with PPI and professional practice groups	Patient perspective Skills & personal development Ongoing dialogue Involvement at all project stages	*	*	NIHR
Ramaswarmy 2020 [37]	University United Kingdom/global	To describe how concepts drawn from the field of implementation science can be used to improve the consistency and quality of Enhanced Recovery After Surgery (ERAS) implementation	Critical reflection	NA	Surgery	Conceptual Overview of EBCD concepts in the implementation of ERAS service development	EBCD Patient as co-creator of design process and services	NR	*		NR^b^
Raynor 2020 [38]	University Leeds Bradford	To examine the feasibility and acceptability of health service researchers co-leading EBCD in multiple healthcare settings as part of intervention development	Critical reflection Patients, family/carers, health processionals	“Improving the Safety and Continuity of Medicines management at Transitions of care” (ISCOMAT) was used as a case study	Health services research	Methodological Feasibility, acceptability and barriers to intervention development using EBCD	EBCD Interviews Patient & staff feedback events Joint feedback event Co-design group meetings	Including all perspectives Involvement at all project stages Respecting & valuing all contributions	*		NIHR
Rousseau 2019 [39]	University United Kingdom	To describe and understand the views and experiences of developers and stakeholders about how design occurs in health intervention development	Qualitative reflection	NA	Health services research	Methodological How design occurs in complex health intervention development	Co-design	NA	*		Medical Research Council
Young 2019 [40]	University NHS Trusts Leicester Lancashire	To describe the process used to co-produce progression criteria for a feasibility study of a complex health intervention	Qualitative Patients, clinicians, academics	NA	Health services research	Methodological Outlining method of co-producing “progression criteria” within feasibility studies	Co-production Individual discussion groups Mixed discussion groups (idea generation, voting, ranking, discussion)	Sharing of power Respecting & valuing all contributions Including all perspectives Training and support		*	NIHR

Author (year)	Lead organisation /location	Aim	Study design /stakeholder type	Intervention reported	Health specialty	Outcomes of interest	Type of co-production used/methods	Co-production features/principles	Reports research or methodology gaps	Reports policy/practice implications	Funder or source of funding
(ii) Co-production in applied health research (*N* = 64)											
Intervention development or evaluation (*n* = 34)											
Ali 2018 [41]	University N England	To develop a simple health literacy intervention aimed at supporting informed reproductive choice among members of UK communities practising consanguineous marriage	Qualitative Researchers, product designer, community leaders, religious leaders, lay members, health professionals	Information leaflets/material to enhance health literacy	Public health—reproductive health	Output Information leaflets; audio and video clips on a local NHS website link	Co-design Interviews, focus groups (with vignettes), participatory workshops	Including all perspectives Respecting & valuing all contributions Ongoing dialogue Involvement at all project stages	*		NHS Leeds NIHR
Beal 2019 [42]	For-profit company United Kingdom	To share an approach to improve the quality of care and services in a secure mental health setting by valuing the contribution of family and friends	Quality improvement Health workers, family and friends of people with mental ill health	Carer toolkit	Mental health	Methodological Ways to carry out co-production with family and friends; lessons learned Output Co-produced carer toolkit	Co-production Workshops Co-presentation of outputs	Working “with” families and friends	*		NR
Bielinska 2018 [43]	University NHS Trust London	To co-design an interview topic guide to explore healthcare professionals’ attitudes towards future care planning with older adults in hospital	Qualitative Patients, carers, health professionals	An interview topic guide	Older people	Methodological Benefits of multi-professional, patient and carer involvement in co-design Impact Understanding of hospital-based anticipatory decision-making	Co-design Patient and carer panel	NR			NR
Best 2019 [44]	University Swansea/global	To investigate the use of innovative teaching methods and share a four-step model, to promote the use of co-production in mental health practice	Qualitative Lecturers, undergraduate and postgraduate students in nursing and social work, mental health service users	A four-step model to help develop co-productive teaching methods	Mental health	Output A four-step model to help develop co-productive teaching methods which ultimately empower students and service users	Co-production World café	Building relationships Respecting & valuing all contributions Joint decision-making Sharing of power	*	*	NR
Bolton 2020 [45]	University London	To evaluate a community-organized health project by comparing results from two different designs—researcher-controlled and community-controlled	Evaluation Communities, health professionals, academics	Community-organized health project (Parents and Communities Together)	Public health/maternal and child	Methodological Challenges of using researcher-controlled designs to evaluate community-led interventions Differences in results of the two evaluations	Co-production Social support meetings Health education workshops	Reciprocity Building relationships	*		Guy’s & St Thomas’ charity NIHR
Chisholm 2018 [46]	NHS Trust London	To explore the processes that facilitated EBCD with carer involvement	Case study Service users, carers, health professionals	Family and carer EBCD project	Mental health	Impact Perceptions of the project and participation in it; factors that help and hinder progress; theoretical model of key processes	EBCD Process-mapping Videos Co-design groups using role play	NR	*	*	No funding
de Andrade 2020	University Scotland	To explore how asset-based approaches and co-production could be used to engage “hard-to reach” communities	Qualitative Community members, professional stakeholders (government, voluntary & third sector)	Asset-Based Indicator Framework	Health research	Impact Developed and critiqued participant-led frameworks for asset-based approaches to address health inequalities; co-production with Black minority ethnic groups	Co-production Community-based participatory action research Action-research workshops with professionals and community members & professionals Video Reflexive journals	NR	*	*	ESRC (Economic and Social Research Council)
Dent 2019 [47]	NHS Trust Kent	To examine the value of appreciative inquiry (AI) methodology in enabling co-productive work within mental health service development	Case study	Appreciative inquiry	Mental health services	Impact Description of the use of AI; observations on its use in mental health service improvement	Co-production The application of AI in co-production	NR	*	*	NR
Eades 2018 [48]	Charity NHS Mental Health Trust Berkshire	To quantitatively measure any impact that independent mental health advocacy (IMHA) support had on patients’ self-determination	Evaluation Patient volunteers resident in hospital	An IMHA service	Mental health	Health Psychological well-being and self-determination; autonomy, competence and relatedness Output Co-produced questionnaire	Co-production Focus group with patient volunteers	NR			NR
Farr 2019 [49]	University Bristol	To investigate the feasibility and acceptability of the pilot implementation of a co-designed care pathway tool (CPT) in professionals’ practice to co-produce care plans and enable efficient working	Qualitative Service users, mental health practitioners, service development staff	CPT	Mental health	Impact On normalization process theory constructs Output an electronic CPT	Co-design Iterative co-design and testing	Used co-production principles (not elaborated) Training and support		*	NIHR Otsuka Health Solutions
Faulkner 2021 [50]	Independent service user University London	To inform researchers, practitioners and policy-makers about the value of user leadership in co-productive research with practitioners, particularly for a highly sensitive and potentially distressing topic	Observational Service users, practitioners, academics	User-led study “Keeping Control”	Mental health	Conceptual Highlights the importance, achievements and benefits for all people involved in co-producing research Methodological Explores the methodological aspects of a user-led study investigating service user experiential knowledge	Co-production User-led interviews with service users Focus groups with practitioners Social media discussion Stakeholder sense-making event	Shared aims and values Joint decision-making Agreed co-production working principles (not elaborated)		*	NIHR
Gartshore 2018 [51]	University London	To explore the implementation and impact of a service user-led co-design intervention to improve user and staff experience on an adult acute psychiatric inpatient ward	Evaluation (mixed-method) Service users, clinical and managerial ward staff	EBCD quality improvement intervention on a mental health admission ward	Mental health	Methodological Awareness of EBCD Impact Challenges and benefits of co-design; factors contributing to implementation of EBCD	EBCD Observations and interviews with staff Videos of service user narratives Staff and joint staff & service user feedback events	NR	*		NR
Gault 2019 [52]	University London	To co-produce consensus on the key issues important in educating mental healthcare professionals to optimize mental health medication adherence in Black, Asian and Minority Ethnic (BAME) groups	Qualitative Service users, carers, student nurses	Educational intervention for mental healthcare professionals	Mental health	Impact Users able to challenge original intention of the study Health Perceptions of factors enabling or disabling medication adherence Output Consensus on content and delivery of an educational intervention for health professionals	Co-production Interviews with service users & carers Consensus workshop with users & carers	NR	*	*	Health Innovation Network South London
Giebel 2019 [53]	University Liverpool	To assess the extent of public involvement, experiences of public advisers and resulting changes in the dissemination of the North-West Coast household survey	Qualitative Public advisors, partner in local authorities and NHS Trusts, academics	Dissemination of a household health survey	Health research	Methodological Extent of public involvement; lessons for improving public involvement; experiences of involvement in dissemination of survey findings Impact Improved dissemination of survey results	Co-production Focus group discussion Co-production workshop with public advisers, partners from local authorities and NHS Trusts, academics	Support Respecting & valuing all contributions Transparency	*	*	NIHR Wellcome Trust
Girling 2019 [54]	University Newcastle	To explore how young people presenting to youth justice services describe and understand their mental health needs, and to explore how EBCD could be applied to facilitate service developments	Qualitative Service providers, academics	EBCD intervention with young people who offend	Mental health	Methodological Challenges in EBCD; effects of including first-hand experiences; shared experiences of challenges among researchers applying EBCD	EBCD Interviews with staff and academics	NR	*	*	NIHR
Halsall 2019 [55]	NHS Trust Lancashire	To address the challenges of co-production through use of social media by creating a Facebook forum for discussion and consultation	Quality improvement Service users, health professionals	Closed Facebook forum for members with either lived or professional experience of perinatal mental health issues	Mental health	Methodological Perceptions of participation in the forum & how it shaped service developments	Co-design Facebook forum to discuss service developments	NR			NR
Horgan 2018 [56]	University Ireland/global	To develop an understanding of the potential contribution to mental health nursing education by those with experience of mental health service use	Qualitative	Co-produced mental health content for nursing students	Mental health	Methodological Views on service user involvement in mental health nursing education; value of lived experience in improving mental health nurses education	Co-production Focus groups	Involvement at all project stages	*		Erasmus+
Horgan 2020 [57]	University Ireland/global	To develop standards to underpin expert-by-experience involvement in mental health nursing education based on lived experience of service use	Qualitative Service users, nursing academics	Standards for co-producing mental health nursing education	Mental health	Methodological Enablers and barriers to involving experts by experience in nursing education; framework to support this involvement	Co-production Focus groups Consensus-building discussion	Involvement at all project stages Joint decision-making Continuous reflection		*	Erasmus+
Hannigan 2018 [58]	University Ireland	To use a participatory health research approach to involve communities in examining the implementation of ethnic identifiers in primary care	Qualitative Researchers, community members, decision-makers	Ethnic identifiers in primary care	Public health	Health Understanding and addressing inequalities among minority and majority ethnic groups in access to healthcare and health outcomes	Co-construction Co-creation Participatory learning and action techniques Focus groups Interviews	Involvement at all project stages Joint decision-making Sharing of power			Health Research Board
Hundt 2019 [59]	University Warwick	To critically analyse the co-production of knowledge on healthcare with members of the public attending two research-based plays that were followed by post-show discussions with expert panellists	Evaluation (mixed-method) Academics, health and social care professionals, service users, theatre directors and writers	Two research-based plays on decision-making towards the end of life (*Passing On*) and mental health (*Cracked*)	Applied health research	Impact Effect of dialogue between different stakeholders in co-production of knowledge; understanding of the health topics; views on inclusion of service users’ perspectives and experiences; enhanced public engagement	Co-production Interviews Developmental drama workshops Discussion and debate	NR	*		University, ESRC, Wellcome
Leask 2019 [60]	University Glasgow/global	To identify a key set of principles & recommendations for co-creating public health interventions	Case study End users, stakeholders, researchers	To identify a key set of principles and recommendations for co-creating public health interventions	Public health	Methodological Development of a framework of principles to facilitate co-creation Output Five key principles: framing the aim of the study; sampling; manifesting ownership; defining the procedure; and evaluating (process and intervention)	Co-creation Action research reflective cycles conducted electronically and face to face	Co-creation principles agreed	*	*	No funding
Litchfield 2018 [61]	University Birmingham	To use co-design principles to source, implement and evaluate improvements in the blood test and result communication process in United Kingdom primary care	Evaluation (mixed-method) Staff and patients	Interventions to improve the blood testing and result communication process	Primary care	Methodological Situational and organizational barriers; participant experiences and influence on service improvement	Co-design Focus groups with staff and patients mixed	Co-design principles mentioned (not elaborated)	*	*	NIHR
Lloyd-Williams 2019 [62]	University Liverpool	To evaluate stakeholder involvement in the process of building a decision support tool	Observational NHS commissioners, GPs, local authorities, academics, third-sector and national organizations	NHS Health Check Programme	Health services research	Impact Stakeholder views, experiences, expectations	Co-production Iterative workshops e-platform	Co-production principles mentioned (not elaborated)			NR
Luchenski 2019 [63]	University London	To explore involving nonacademic communities in co-developing research priorities, with particular emphasis on traditionally excluded groups	Qualitative People with experience of exclusion, representatives from the NHS, charities, national, regional and local government and academic institutions	An advocacy agenda for Inclusion Health	Health inequalities	Methodological Making PPI more inclusive to excluded groups	Co-production One-day event with inclusive, participatory and consensus-building activities	Co-production approach mentioned (not elaborated)	*	*	University Grand Challenges
Marent 2018 [64]	University Brighton/global	To use a reflexive approach to evaluate a co-designed mHealth platform for HIV care	Evaluation Clinicians, patients	A digital HIV/AIDS support & self-management platform	HIV/AIDS	Conceptual How a reflexive approach can generate understanding & anticipation towards a new intervention Output An mHealth platform for health monitoring	Co-design Peer-led co-design workshops Interviews	NR	*	*	EU
Miles 2018 [65]	University London	To discuss how “slow co-production” is an underused but valuable tool for co-production in healthcare design	Qualitative Young people with sickle cell and their carers, healthcare providers	This Sickle Cell Life: co-produced research to improve child-to-adult sickle cell patient care transitions	Health services research	Methodological How slow co-production, with content led by priorities of patient, enables deeper insights and better service improvement	Co-production Repeated interviews & participant diaries with young people Interviews with healthcare providers	Involvement at all project stages	*	*	NIHR
O'Connor 2020 [66]	University Edinburgh	To explore the perspectives of stakeholders involved in co-designing a mobile application with people with dementia and their carers	Qualitative People with dementia and their carers, a museum, a software company, and an NHS Trust	App to support communication between carers and people with dementia (Innovate Dementia)	Older people	Methodological Experiences of being involved in co-design Impact Value of the health app Health Health and well-being benefits	Co-design Living laboratories Interactive co-design workshops	NR	*	*	Burdett Trust
Pallensen 2020	University Ireland	To evaluate stakeholder experiences of the co-design process	Qualitative Researchers, healthcare providers, a patient representative	Team-based Collective Leadership and Safety Culture (Co-Lead) programme to improve performance and patient safety	Health services research	Methodological Expectations for and experiences of the process; positive aspects and challenges; decision-making process; learning and impact	Co-design Workshops involving researcher inputs, experience-sharing and co-design	Collective leadership	*	*	Irish Health Research Board
Patel 2018 [67]	Public Health England London	To pilot co-production, delivery and evaluation of oral care training for care home staff	Qualitative Care home managers, residents and family members	Oral health training DVD for care home staff; training resources; oral care support sessions	Older people	Impact Oral health knowledge; views on training; areas for improvement	Co-production Action research Questionnaire and interview with care home managers Informal discussions with residents and family	Including all perspectives Respecting & valuing all contributions	*		NR
Ponsford 2021 [68]	University London	To describe the approach to co-producing two whole-school sexual health interventions for United Kingdom secondary schools	Qualitative Researchers, secondary school staff and students, youth and policy and practitioner stakeholders in sexual health	Positive Choices aimed at preventing unintended teenage pregnancy Project Respect aimed at preventing dating and relationship violence and sexual harassment in schools	Adolescent health	Output Two teacher-led, classroom-based sexual health interventions Methodological Description of stakeholder consultation to inform intervention development; challenges and dilemmas encountered; extent of co-production	Co-production Consultation meetings with students and staff using small group working Meetings with youth group Meetings with policy-makers & practitioners	NR	*	*	NIHR
Rodriguez 2019 [69]	University Dundee	To develop co-design, implement and evaluate a series of oral health workshops with young people experiencing homelessness	Qualitative Nongovernmental organization managers and staff, practitioners, homeless young people	Eight workshops raising health awareness, including oral health, mental health, substance abuse and healthy eating	Oral health	Impact Changes in behaviour, knowledge, health literacy, engagement with service providers Methodological Workshop experience; common positive elements of workshops	Co-design Action research Meetings Workshops Interviews	Mutual trust Joint decision-making	*	*	Scottish Government and Health Service Board
Scott 2020 [70]	University Dundee	To co-design and evaluate an animated film promoting oral health	Evaluation (mixed-method) Parent–child dyads	Short, animated film promoting oral health	Oral health	Impact Oral health knowledge Feedback on film content, messages and visuals Output Short film promoting oral health	Co-design Workshops including an activity sheet, ranking exercise and feedback on storyboards and animated films Interviews with parents Questionnaire	Co-design and co-creation strategies mentioned (not elaborated)	*	*	Public Health England
Tribe 2019 [71]	University London	To discuss examples of co-produced mental health training, working with refugee or migrant community groups	Qualitative Academics, practitioners, community workers	Training for staff in a United Kingdom refugee community centre Training workshop in Sri Lanka to develop skills for coping while living in a war zone	Mental health	Health knowledge and well-being Impact Contribution of co-production and partnership working to knowledge and practice	Co-production Meetings Workshop Interviews	NR	*		NR
Whitham 2019 [72]	University Lancaster	To discuss risks and benefits of co-designing tools for use by practitioners and implications for sustainability and impact of co-design initiatives	Case study Health and social care staff and service uses	Tools to improve difficult conversations in health and social care practice (Leapfrog tools)	Health and social care	Output Conversation tools for use by practitioners Impact Risks and benefits of co-designing tools for use by practitioners; sustainability of co-design initiatives	Co-design Participatory action research Tool co-design activities Sharing activities to disseminate tools Evaluation activities	Including all perspectives Sharing of power	*	*	Arts and Humanities Research Council
Systematic or evidence reviews or overviews (*n* = 10)											
Ball 2019 [73]	Nonprofit organization Cambridge	To review the evidence base on patient and public involvement (PPI) in research, in order to determine what is known in and where there are gaps	Rapid evidence review	NA	Health services research	Impact Challenges to PPI Impact of PPI	Various	NA		*	THIS Institute
Barnett 2020 [74]	University United Kingdom/global	To discuss key challenges relating to interdisciplinarity, epidemiology, participatory epidemiology, including the meaning of co-production of knowledge	Review	NA	Public health—One Health	Conceptual Understanding what co-production means in relation to knowledge production in One Health Methodological Challenges in doing co-production working across disciplines and cultures	Co-production	NA	*		Biotechnology and Biological Sciences Research Council UK Research and Innovation
Bench 2018 [75]	University London	To synthesize current evidence on best practice for PPI within critical care	Scoping review	NA	Critical care	Impact Levels of involvement Involving critical care patients Barriers to/facilitators of PPI	Various	NA	*	*	NIHR
Connolly 2020 [76]	University W Scotland	To learn how co-production and co-creation is understood, implemented and sustained within the health and social care system in Scotland	Rapid evidence review	NA	Health & social care services	Impact Impacts pf co-production and co-creation on service improvements; evidence of effectiveness; barriers to & facilitators of co-production; sustainability of co-production and co-creation	Co-production Co-creation in health & social care services	NA	*	*	Scottish Improvement Science Collaborating Centre (SISCC)
Green 2020 [77]	University Global	To examine the use (structure, process and outcomes) and reporting of EBCD in health service improvement activities	Systematic review	NA	Health services research	Methodological Use of EBCD (structure, process, outcome) Reporting of EBCD in health service improvement projects	EBCD	NA	*	*	University
Halvorsrud 2021 [16]	University NHS Trust London	To investigate the effectiveness of co-creation/production in international health research	Systematic review	NA	Public health	Impact Effects on health behaviours, service use and physical health Methodological Process elements in effective projects	Co-creation Co-production	NA	*	*	Lankelly Chase Foundation
Pearce 2020 [78]	University United Kingdom/Australia	To propose a new definition of co-creation of knowledge based on the existing literature	Literature Review	NA	Health research	Conceptual New definition of co-creation of new knowledge for health interventions	Co-creation	NA	*	*	Australian government scholarship
Sherriff 2019 [79]	University Brighton	To determine what is known about healthcare inequalities faced by LGBTI people, the barriers faced whilst accessing healthcare, and by health professionals when providing care, and examples of promising practice	Rapid reviews co-produced with LGBTI people	NA	Health inequalities	Health Inequalities and barriers to accessing healthcare	Co-production	NA			European Parliament
Slattery 2020 [15]	University Global	To identify the current approaches to research co-design in health settings and evidence of their effectiveness	Rapid evidence review	NA	Health services research	Conceptual Co-design approaches and activities Methodological Effects of existing co-design approaches	Co-design	NA	*		Transport Accident Commission
Tembo 2019 [80]	University Southampton	To explore whether and how the public can be involved in the co-production of research commissioning early on in the process	Literature review	NA	Health research	Conceptual Whether and how public can be involved in research commissioning Impact Challenges to public involvement in early phase of applied health research	Co-production		*	*	NIHR
Critical reflection or opinion (*n* = 20)											
Beresford 2019 [81]	University Essex	To put public and user involvement in health and social care into broader historical, theoretical and philosophical context	Commentary	NA	Health research	Impact Identifies four key stages in development of public participation in health and social care; barriers/challenges to public participation; successful participation in learning & training and in research knowledge production	Co-production	NA	*	*	NR
Dowie 2018 [82]	University London	To elaborate the implementation of apomediative (“direct-to-consumer”) decision support tools—used by individuals to help make healthcare decisions for themselves—through the technique of multi-criteria decision analysis	Commentary	NA	Public health	Conceptual Importance of shared decision-making between patient and professional about healthcare, through the use of decision support tools	Co-creation of health by patient and health professional	NR			No funding
Green 2019 [13]	University Essex	To offer a global and provocative perspective on participation as emancipatory and reformative vs participation as a servant to neoliberal capital forces	Commentary	NA	Health services research	Conceptual Theoretical critique of participation in healthcare Methodological Evidence about the potential for participation and co-production; realities and challenges in achieving co-production; ways to facilitate co-production	Co-production	NA	*	*	NR
Fletcher 2020 [83]	University Edinburgh	To analyse how health research regulation is experienced by stakeholders in the United Kingdom	Delphi survey	NA	Health research (regulation)	Impact Direct experience of health research regulation by researchers, regulators and experts	Co-production Mentioned as an outcome not a process	NA	*		Wellcome Trust
Hoddinott 2018 [84]	University Scotland/United Kingdom	To outline how researchers can involve patients in funding applications and pitfalls to avoid	Opinion	NA	Applied health research	Conceptual Definitions of patient and public involvement, co-design, co-production Methodological How to involve patients in research; opportunities and pitfalls	Co-production Co-design	NA	*	*	No funding
Kaehne 2018 [85]	University Lancashire	To outline current thinking on co-production in health and social care, examine challenges in implementing genuine co-production	Commentary	NA	Health and social care	Conceptual Definitions and explanations of co-production Methodological Establishing parameters of a co-production model; barriers to co-production in health and social care	Co-production	NA	*	*	NR
Kislov 2018 [86]	University Manchester	To explore different definitions and types, tensions and compromises, and implications of, analyse the factors influencing, and share personal experiences of co-production	Qualitative/participatory	Interactive workshop	Applied health research	Conceptual Definitions and types of co-production of evidence in applied health research Methodological Tensions and compromises of doing co-production; factors influencing processes and outcomes of co-production	Co-production	NA	*	*	NR
Kislov 2019 [87]	University Manchester	To explore the processes, mechanisms and consequences of co-production between researchers and practitioners as an approach facilitating the implementation of research in healthcare organizations	Case study Producers and users of applied health research	Four applied health research projects	Applied health research	Conceptual Definition of co-production approaches Methodological Compromises and negative consequences of co-production of applied health research	Co-production	NA	*	*	NR
Lambert 2018 [88]	University London	To explore the development of co-production and service user involvement in United Kingdom university-based mental health research	Commentary	NA	Mental health	Conceptual How co-production of mental health policy, practice and research is conceptualized Methodological Implications of co-production; reflection on the practice of research co-production (process, barriers, outcomes)	Co-production	NA	*	*	NR
Langley 2018 [89]	University Sheffield	To explore the different domains of influence of collective making from a knowledge mobilization perspective	Commentary	NA	Health and social care	Conceptual How the “collective making” co-design model contributes to co-creation of knowledge	Co-creation Co-design	NA	*	*	NIHR
Lignou 2019 [90]	University Oxford	To describe how a co-produced public health intervention was developed	Commentary	NA	Mental health	Conceptual Explains the application of the concept of co-production to mental health research in four iterative steps	Co-production	NA	*	*	NIHR Wellcome
Metz 2019 [91]	University London/US	To draw out the learning and reflect on the wider co-creation literature and debates	Opinion	NA	Health services research	Conceptual Clarifying and characterizing the use of “co-creation”	Co-creation	NA	*		NR
Norton 2019 [92]	University Ireland	To give guidance on how to implement co-production within Irish mental health services	Opinion	NA	Mental health	Conceptual Definitions, types, principles and models of co-production; barriers to co-production; how to implement co-production	Co-production	NA	*	*	NR
Palumbo 2018 [93]	University Europe	To conceptually explore the risks of value co-destruction in the patient–provider relationship and suggest a theoretical framework containing implementation issues of health services’ co-production	Commentary	NA	Health services research	Conceptual Definition and distinction between individual and organizational health literacy Output Framework of factors for effective health services co-production—including individual and organizational health literacy	Co-production	NA	*	*	NR
Realpe 2018 [94]	University Coventry	To establish a working definition of the co-production of health	Commentary	NA	Health and social care	Methodological Model of the co-production of health in consultations Skills of clinicians and patients, and the context and outcomes of co-productive consultations	Co-production	NA	*		NR
Rose 2019 [95]	University London	To examine the concept and practice of co-production in mental health	Commentary	NA	Mental health	Conceptual Historicizing co-production Methodological Context of co-production; positionality and co-production; privilege in knowledge generation	Co-production	NA	*	*	Wellcome
Smith 2020 [96]	University Newcastle	To examine how Lean methods can be implemented and used to engage stakeholders in defining value and systems and processes in healthcare	Commentary	NA	Health services research	Methodological Structured methods for co-production engaged stakeholders to articulate their own value perspectives	Co-design	NA	*		NR
Syed 2019 [97]	Government Global	To outline a framework for facilitating co-creation of public health evidence	Commentary	NA	Public health	Methodological Definition of co-creation; barriers and facilitators in use of public health evidence Output Evidence-informed public health conceptual framework	Co-creation	NA			NR
Thompson 2020 [98]	University Edinburgh	To describe what form co-production is taking and why in the context of NHS Scotland	Commentary with case study	NA	Health services research	Methodological Examples of co-production within healthcare in Scotland Conceptual Co-production in governance arrangements	Co-design Co-governance	NA	*	*	No funding
Wolstenholme 2019 [99]	Professional clinical association	London	To discuss what co-production is and the impact it can have by drawing on a Twitter chat on co-production and management of acute and long-term stroke	Opinion	NA	Acute care	Methodological Conditions for co-production to happen; activities that support co-production and co-creation; involvement of creative practitioners to improve co-creation process	Co-production Co-creation	NA	*	NR

*NA* not applicable, *NHS* National Health Service

^a^Co-production principles and features as defined by NIHR (https://www.learningforinvolvement.org.uk/?opportunity=nihr-guidance-on-co-producing-a-research-project)

^b^NR not reported

* Indicates when a paper reports research or methodology gaps and/or policy/practice implications

In column 2, the underlined text highlights the type of organisation. In column 8, the underlined text highlights the type of co-production used

Papers reporting co-production in applied health research (*N* = 64) included intervention development or evaluation studies (*n* = 34), systematic, scoping or rapid evidence reviews or literature reviews (*n* = 10) and critical reflection or opinion pieces (*n* = 20). Most studies describing intervention development were qualitative and concerned co-designing or co-producing research methods or tools, or exploring the feasibility or acceptability of co-produced knowledge or service improvements. Evaluations reported on the mechanisms, approaches and forms of co-produced research projects, or measured impact or effects of co-produced interventions or projects. The systematic, scoping and rapid evidence reviews summarized best practice, definitions, implementation and sustainability, reporting and effects of co-produced research. The opinion and reflection papers tended to summarize historical or theoretical perspectives on co-production or user involvement in research, as well as outlining current thinking, literature and debates relating to co-designed or co-produced research, while others offered opinion on how to realize co-production and tips for effective co-production of services and research.

The included studies represent a broad spectrum of health specialities or disciplines. For those reporting co-produced complex interventions, many of the reviews and opinion pieces related to health services research or biomedical research, while the intervention development studies were situated in public health (*n* = 2), acute and intensive care (*n* = 1), addiction and substance misuse (*n* = 1), older people (*n* = 1), chronic illness (*n* = 1), primary care (*n* = 1), social care (*n* = 1), maternal health (*n* = 1) and mental health (*n* = 1). The studies reporting co-production in applied health research were related to health services research (*n* = 21), or were conducted within specific specialities such as mental health (*n* = 19), public health (*n* = 7), health and social care (*n* = 4), older people (*n* = 3), critical or acute care (*n* = 2), health inequalities (*n* = 2), oral health (*n* = 2), primacy care (*n* = 1), HIV/AIDS (*n* = 1), chronic illness (*n* = 1) or adolescent health (*n = *1).

A range of outcomes were reported across all studies including conceptual (e.g. defining or explaining some aspect of co-production), methodological (e.g. focused on the process of designing or carry out co-production), impact (e.g. challenges, barriers and facilitators of co-production, acceptability, cost or effectiveness of co-produced research) and health (e.g. impact of co-produced interventions on health outcomes). Many studies resulted in tangible outputs or products including toolkits, models, frameworks or principles (see Table 2). Five studies concerned with applied health research described co-production as a means for “knowledge mobilization” or “knowledge transfer”, including co-produced dissemination activities [53], public engagement for better understanding of health topics [59], co-production for facilitating research implementation [87], use of co-design for knowledge mobilization [89] and co-creation of public health evidence [97].

Overall, nine studies (47%) reporting on co-produced complex interventions and 12 (19%) of those reporting co-production in applied health research were funded or supported by the NIHR. Other funding sources for studies of co-produced complex interventions included PhD studentships or fellowships (*n* = 3), National Health Service (NHS) Trusts (*n* = 1), Medical Research Council (*n* = 2) or National Science Foundation (*n* = 1), or the funding source was not reported (*n* = 3). Funders of co-production in applied health research included Wellcome (*n* = 4), charities (*n* = 4), Scotland or Ireland health boards (*n* = 4), European Union or Erasmus+ (*n* = 4), United Kingdom Research Councils (*n* = 3), university/Grand Challenges (*n* = 2), other government funding (*n* = 2) and single-study funding by an NHS Trust, an Academic Health Science Network (AHSN), Public Health England or private/commercial funding. Five applied health research studies did not receive funding and 23 did not report the funding source.

In most studies reporting on the development or evaluation of co-produced interventions, the lead organizations were universities (8/10 complex interventions and 27/34 co-produced applied interventions). Very few were led or co-led by NHS Trusts (2/10 complex interventions and 4/34 applied interventions) [23, 29, 43, 46, 47, 55], one study was led by a community organization [26], and two co-produced applied interventions were led by independent service users or service user charities [48, 50].

### Conceptualization and implementation of co-production

Fifty-five papers referred to co-production either independently or in conjunction with other terms such as PPI/E, co-creation or co-design (see Additional file 2). Twenty-three papers were concerned with either co-design or EBCD, 12 used the term co-creation, and 10 mentioned PPI/E. Sixty-eight papers reported their research as a single methodology (e.g. co-production, co-design, EBCD or co-creation), with the remaining 16 using a combination of these terms to describe their work (e.g. co-production/co-creation, co-production/PPI, co-production/co-design or co-creation/co-design).

Some papers were very explicit in the definition of their chosen term, whereas others opted to describe the term using references from pre-existing literature. A commonly referred to definition was that of PPI as defined by INVOLVE, a national advisory group for PPI: research being carried out “with” or “by” members of the public rather than “to”, “about” or “for” them” [100]. In some instances a distinction was made between PPI/E and other co-activities based on the level of “active involvement” or the presence of a shared-power dynamic, with PPI/E being seen as a more passive or advisory role with a lower share of power and control [34, 88, 95]. A number of authors, however, seemed to use the two terms interchangeably [53, 75, 81].

Co-production was the most widely used term, referring to both the co-production of research and the co-production of services. The concept of shared power was widely used when describing co-production and what it means to health research or service development [42, 52, 101]. In these definitions, and indeed many others, co-production implied the involvement of a variety of stakeholder groups (e.g. services users, charity representatives, healthcare professionals and academics) in multiple stages of the research process. Others, however, used co-production as an umbrella term encompassing all aspects of additional stakeholder involvement whether that be throughout the process or in a single stage of the research cycle. In their rapid review evaluating hospital tools and services that had been co-produced with patients, Lim et al. included “co-production (e.g. co-production, co-design… [and] co-creation)” in their search terms [32], which highlights its use as a catch-all term.

Co-design was usually used to refer to stakeholder involvement in the design process of user-friendly tools, interventions or initiatives. Emphasis was placed on the value that “experts by experience” (e.g. patients, services users or clinicians) can bring to the design process as equal partners, beyond user involvement or consultation [102]. Stakeholder groups involved in these co-design projects included patients, carers, healthcare professionals, service users, local people and software or technology developers [13, 43, 46, 49, 51, 54, 61, 103]. Another frequently mentioned term was EBCD, which was defined by Chisholm as “a service design strategy that facilitates collaborative work between professional staff and service users toward common goals” in every stage of the design process [46]. EBCD appears to be more often applied to service development, while co-design is more often referred to in research.

Where it was possible to discern how the concepts were enacted, the type of methods reported in papers describing co-production or co-design included individual interviews, group workshops, reflection and discussion meetings, focus group discussions, social media forums, surveys, or a mix of these activities [47, 48, 52, 55, 62, 63, 84] (see Table 2). While some papers described specific activities and participatory approaches used in co-design or co-production workshops or meetings [25, 30, 70, 72, 104], most did not elaborate on their methods. In studies describing intervention development or evaluation, we looked for reference to principles of co-production or co-design (defined by the NIHR^1^) and how they were enacted. Of the studies reporting development of complex interventions (*n* = 10) and studies reporting development of applied interventions (*n* = 34), the principles described most frequently as key features of the projects were “including all perspectives”, “respecting and valuing all contributions”, “joint decision-making” and “involvement of stakeholders at all project stages”. Very few papers referred explicitly to “sharing of power” among stakeholders, or the principles of “reciprocity” and “building and maintaining relationships”. Fourteen of the applied research intervention studies did not mention co-production principles at all, and seven stated that co-production or co-design principles or approaches were used or agreed on, but specific features were not described (see Table 2).

Most studies reported on the stakeholder groups involved in co-producing interventions (Table 2), and most often these were combinations of academics/researchers, patients/carers/service users and health professionals/practitioners. Where project specialties or specific focus dictated, family members, friends, community members and representatives of other organizations were included as stakeholders. The studies did not report on stakeholder criteria, how stakeholders were chosen or the qualifications required to participate in co-production. A number of papers described having involved stakeholders as early as possible in the research process, with some even initiating patient involvement before drafting their funding application [36].

Co-creation and co-production appear to have considerable crossover in the literature, with similar definitions being used for both terms. In one report, Connelly et al. agreed that their review of the literature found that “co-production and co-creation are largely very similar”, but thought that co-creation represented a more prolonged involvement of stakeholders “throughout the process of programme design, development, implementation and evaluation (not just at the programme development and design stages)”. A similar idea was shared by Hannigan, who used co-creation to describe the involvement of additional stakeholders in all aspects of the project, namely “co-design of the research protocol, project governance, collaborative data interpretation and disseminating findings” [58]. As with co-production, co-creation has also been used as an umbrella term to describe the involvement of wider stakeholders in healthcare research [16]*.*

### Lessons for the practice of co-production

Content analysis of information extracted from the papers revealed several recommendations for the practice of co-production and more strategic requirements for co-production to become more established and embedded in organizational structures: (1) capacity to implement co-produced interventions, (2) skill set needed for co-production, (3) multiple levels of engagement and negotiation, and (4) funding and institutional arrangements for meaningful co-production.

#### Capacity to implement co-produced interventions

Papers concerned with co-production of applied health research emphasized building capacity to adapt and absorb the changes brought about through co-production and co-creation, and fostering a cadre of implementation-savvy researchers who can “do implementation” was considered vital [80, 86, 87]. Others highlighted middle managers as “critical catalysts” for strategic and operational impact of co-produced interventions, and frontline staff as key enablers or “active agents” of change [29].

#### Skill set needed for co-production

The papers also emphasized the skills required of researchers for co-production, which authors felt lay outside the typical academic or researcher skill set [29]—for example, collaborating with diverse stakeholders, negotiation skills [90], good persuasive communication [29, 46], managing expectations [29], prolonged involvement with service users and other stakeholders, and flexibility in maintaining relationships [30, 104] and showcasing outcomes [46]. One paper recognized the difficulty of doing co-production as a mandated activity and acknowledged that not everything has to be co-designed and not everyone will want to occupy this space [35].

#### Multiple levels of engagement and group negotiation

Many papers reporting co-produced complex interventions highlighted the importance of multiple levels of engagement (patient or user, practitioner or provider, and policy-makers) as well as multiple levels of experience and values (individual, family, organizational, cultural, political) for a truly participatory process [24, 25, 40, 84, 102]. This was thought to require leadership and careful negotiation of group politics for meaningful and productive discussions [24, 25], a balance of experienced and new co-production contributors and clear boundaries for involvement [84], as well as the ability to navigate the different types of knowledge, experience, research literacy, priorities and perspectives of diverse stakeholder groups involved [40].

With various levels and types of engagement, the need to resolve (inevitable) disagreements was mentioned in several papers. Some highlighted that when disagreement arises between stakeholders and researchers, or when there is conflict between service users, practitioners and organizational perspectives [49], discussion of differences should be encouraged and not regarded as a threat [24]. Similarly, allowing stakeholders to challenge researchers’ intentions and assumptions was encouraged and regarded as beneficial [52]. However, it was recognized that patients and other groups rarely feel able to challenge the hegemony, and this has implications for carrying out co-production and collaborative work with patient groups [61]. Others advocated for early consultation between all stakeholders to mitigate disagreements in relation to the aim, direction and outcomes of co-produced research [26].

#### Funding and institutional arrangements for meaningful co-production

Several papers mentioned that meaningful co-production required certain funding and institutional arrangements [36, 65, 76, 81, 84]. The main concerns were the need for sufficient funding to cover planned co-production activities [84] and to adequately resource co-production to ensure inclusivity, diversity and equality [81]. Others suggested that current funding systems prevent meaningful co-production in the planning stages of research [36] and that there is often insufficient time to establish relationships with patients and other groups [65]. Some papers questioned whether existing institutional arrangements could support the ideals of co-production and manage the tensions that arise [36]. It was also recognized that effective co-production requires changes in academic institutions and scientific practice, specifically to embrace more equal power distribution in the research process and ensure proper governance for co-production [60]. Others called for leadership for co-production to be more embedded in local health and social care organizations [76].

### Themes for future research on co-production

Most of the papers reporting co-produced complex health interventions (16/19), and those relating to co-produced applied health research (50/64), reported research implications of their work, or suggested future directions for research on co-production. Content analysis of information extracted from included studies led to the following themes for future research: (1) who to involve in co-production and how; (2) evaluating outcomes of co-production; (3) the language and practice of co-production; (4) documenting costs and challenges; (5) vital components for co-production.

#### Who to involve in co-production and how

Many of the papers concerned with co-produced applied health research highlighted the need for future research to focus on better ways of involving more diverse groups of service users and stakeholders [31, 63, 68–72, 80, 95, 98]. There were suggestions for research to focus on understanding how to involve different groups and facilitate effective involvement [54, 77]. For example, it remains unknown whether planned activities [99] or less structure [72] allows for better involvement and more successful adoption of co-produced interventions. One questioned whether more extensive input from multiple stakeholders equates to more effective interventions [31].

Other papers relating to co-produced applied research suggested that future research should focus on identifying how best to recruit and involve people in co-production or co-creation [75, 91], specifically to identify which engagement strategies work best and whether different levels or types of engagement suited particular stakeholder groups. Research on how to build trust was also thought to be a priority, since it is the foundation for successful partnerships and co-produced interventions that are more likely to meet needs [91].

#### Evaluating outcomes of co-production

There was considerable discussion in the included papers on complex interventions about evaluating the impact of co-production and co-produced interventions on patient and provider outcomes, and the dominance of certain study designs [30, 32–34, 37, 104]. Concerns included the dominance of qualitative, case study and mixed-method research for evaluation of co-production [33, 34], known for small sample sizes, recruitment bias and weak designs yet strong claims about the effects of co-production [34]. Several authors stated that understanding the causal effects of co-production and disentangling the effects of participant involvement from the effects of co-produced interventions required quantitative techniques including randomized evaluations to promote confidence in causal relationships [30, 34]. Authors also highlighted that outcome evaluations of co-production tend to report positive impacts [34] and moderate to high acceptability, usability and uptake [32], yet the evidence for the effect of co-produced interventions on organizational, patient and health service provider outcomes is limited and the value added uncertain [32]. There is an abundance of research on factors affecting the success of co-production and participation in and experience of co-production, but some suggest not enough focus on broader outcomes (for example, whether co-production is empowering to individuals, increases acceptance of co-production among professionals and policy-makers or increases demand among service users for co-production and co-creation approaches) that really matter to patients and providers [34]. Indeed, some of the reported positive impacts may reflect possible negative outcomes for individuals involved in terms of efficiency and other costs that are rarely reported, for example feelings of pressure and frustration among those taking part in co-production (despite it being empowering) and a lack of time to implement co-production (even though there may be increased appetite for it) [34]. The real costs and benefits and how co-production could be used to produce better outcomes, more efficiently and at less cost are under-researched [34, 37].

Papers reporting applied health research contained much the same discussion, centred on the need for more rigorous evaluation of co-production and its impact on quality, implementation and outcomes of research [31, 45, 59, 66, 85, 90, 91, 95], including longer-term effects on health outcomes derived from co-produced interventions [15, 16, 55] and more “quantitative” research, especially inclusion of comparators as a minimum requirement [45]. One paper specifically mentioned the lack of evidence that co-produced services have led to improved satisfaction or resulted in better quality of care for end users, mainly due to the use of non-comparative study designs [85].

#### The language and practice of co-production

A few papers reporting complex health interventions questioned whether the increased traction of co-production in academic and policy debates had changed attitudes and practice around PPI to produce more authentic collaboration, or whether tokenism persisted [35]. Adaptations to “traditional” co-production approaches were advocated in the complex intervention studies—for example, feasible and acceptable enhancements to the EBCD approach [38]. Others commented that research is needed to compare different co-production approaches, to identify which ways of working for complex health intervention development are likely to maximize creativity and lead to health gains [39].

Many papers reporting applied health research similarly commented on the discrepancy between the language and practice of co-production. Some highlighted the rhetoric and concerns about tokenism of co-production [52, 80]. There were calls to improve and deepen “true” co-production [52] and to develop measures of co-creation in research to indicate the extent to which researchers use the methods they claim to [78]. Comments were also made about the variety of co-production approaches or strategies in use and the adaptation of co-production to different people and contexts [15, 85]. There were also concerns about the diversity of practice and adaptation of co-production and a feeling that more research is needed to understand what conditions of co-production contribute to evidence use and improved outcomes [91], what constitutes effective co-production in which circumstances [80, 85], and at which stages of research co-production is appropriate and useful [15, 90].

#### Documenting costs, challenges, barriers and facilitators

Studies reporting complex interventions reflected on challenges and barriers to co-production [25, 26, 34, 36, 37] and the need for further research to focus on facilitators as well as challenge in co-production. Two studies highlighted facilitators including the use of a “needs analysis” at the first meeting, open questions and subgroups, multidisciplinary debate and a problem-solving approach [25] and a need to identify champions and allies to gain entry and engage stakeholders [29]. There was a view that future research needs to be open to “what doesn’t work” and should be developed based on the learning from previous work [29, 36].

The applied research papers also suggested the need for more open identification, discussion and resolution of challenges in co-produced research [52, 61, 74, 87]. Challenges included inevitable disagreements between researchers and service users [52], and situational and organizational barriers [61] and tensions specifically related to co-production and policy-making (e.g. resolving power issues, high involvement costs and providing incentives to stakeholders to secure buy-in) [87].

#### Vital components for co-production

The included studies concerning co-produced complex interventions pointed towards the need for future work to document and facilitate sharing of best practice. Some suggested that future research should help to understand how co-creation and co-production work in practice [33], and should clarify the concepts and processes to better operationalize co-creation and co-production [34]. Others suggested research to compare different approaches to intervention design to uncover which work best for co-production of complex intervention development and which are most likely to lead to health gains. They also called for future research to clearly and consistently report the methods used [39]. Two studies suggested it was important for future research to document and share best practices in co-production, detailing the components that are vital to the process [26, 37]. One suggested that best practice may only be determined by studying “live examples” of effective co-production strategies [32].

Similar narratives were evident in applied health research papers, with calls for future research to report contextual details that inform the selection of co-production approaches as well as better reporting of activities and processes involved in co-production [15]. Other papers suggested research to explore the mechanisms for optimum success in co-creation, and to determine what factors affect success [16] and which co-production activities are best suited for which research projects or health and social care programmes [85]. One paper reported the development of a framework of principles to facilitate co-creation of local public health interventions [60] and another outlined procedural steps for implementing co-production in mental health services [92].

## Discussion

This scoping review has mapped out the recent literature on co-production in applied health research in the United Kingdom and offers an interpretation of how co-production is being practised and what methodological research questions remain. Co-produced complex interventions were evident across a range of health specialities, from acute and intensive care to public health and surgery. In applied health research, co-production was most apparent in mental health research. The majority of the empirical research we found used observational methods to describe co-production processes and mechanisms or qualitative research to explore stakeholder experiences and perspectives on how co-production can be applied in research or service improvements. The literature also appears to be dominated by commentary or opinion pieces that describe author experiences of co-producing interventions or offer historical or theoretical perspectives of user involvement in research. We found very few empirical studies of the impact or effect of co-produced complex interventions or knowledge within applied health research.

### Implications for co-production practice

The included studies make an important point about co-production ideally involving multiple stakeholders with multiple levels of experience and understanding and of differing values. Because of this rich and diverse participation, highly developed leadership and negotiation skills are often cited as requirements for meaningful co-production. However, this should not imply that one group holds power to facilitate productive discussion or resolve disagreements: power-sharing and managing conflict in co-production is a joint responsibility. Safe space for stakeholders to challenge each other and where all groups feel able to discuss their differences is important. Yet this space should not become so comfortable that it breeds homogeneity, because it is the very diversity of views, experiences, skills and knowledge and the equal importance of all contributions that co-production strives to harness.

That co-produced research requires adequate funding and certain institutional arrangements is an unsurprising finding. There is no doubt, as others have also suggested, that more and reliable funding could overcome some of the barriers [13], such as resourcing activities in the planning stages of research [15] and having enough resources to ensure inclusivity and reciprocity throughout the process [15, 16]. However, our review identified entrenched academic and scientific practices as a potentially greater impediment to progress in co-produced applied health research. Changes required at the individual researcher level such as embracing more equal power-sharing, refining negotiation and communication skills, and managing stakeholder relationships can be developed, but organizational changes such as proper governance and research policies that enable co-production take longer.

### Research implications and gaps in knowledge

The included studies highlighted a need to identify better ways to recruit stakeholders and to facilitate more effective involvement in research, including ways to involve more diverse groups. In applied health research, there are undoubtedly various modes of engagement being tried and tested but probably not reported on. There is clearly a desire to learn from successful projects and teams about specific methods for engagement and flexibility in approaches including whether structured activities or more extensive input from multiple stakeholders makes for better involvement and ultimately better interventions [31, 72, 99]. The research community could do better in terms of reporting this learning. Being explicit about who or what constitutes a stakeholder group is key to identifying how best to involve and collaborate with different groups [3].

The need for more rigorous research on the benefits of co-produced research, the added value of co-produced interventions and their effects on quality of care and satisfaction with services was a dominant finding. Producing this kind of evidence is difficult to do; co-produced processes and outcomes are often context-specific. However, a recently published review that pooled evidence from reviews and primary studies of co-creation from the international healthcare literature found moderate positive effects on immediate health-related outcomes including health service access and health-promoting behaviour, but less evidence on long-term effects [16]. The rather technocratic focus on “more evidence” of effects and impact ignores the democratic rationale for co-production—that it is the right thing to do in principle regardless of the outcomes. Many argue that the endeavour of co-production itself is sufficient to achieve the end goal of collaboration to realize outcomes that would not happen otherwise [3], and quantitative or experimental research to justify its value is unnecessary. Perhaps there is value in regarding co-production as an exploratory “social space” and a generative process rather than a means to deliver impact and outputs [4]. We agree, though, that capturing evidence of the “value” of co-production to participants—in relation to broader outcomes such as equality in power in the research process, empowerment and new skills developed—would be useful, not least to research funders who now expect research to be “co-produced”, as would involving health economists in more creative ways of estimating the cost and cost-effectiveness of co-produced research [34, 37].

Findings relating to the language and practice of co-production in applied health research highlighted the variety in the application of co-production and deviation from “traditional” approaches. Concerns were raised not only about these adaptations, but also about the persistence of tokenistic co-production. We do not think a race to demonstrate what constitutes “true” co-production and authentic co-produced research is what is needed. Rather, we think it is important to be mindful of how divisive this narrative can be. While it is good to aspire to the ideal and hold up the “gold standard” in co-production, the commitment in terms of time, resources or perceived expertise can make this feel unachievable and can put people off [12]. We argue for acceptance of a diversity of approaches to co-production that allows more researchers and others to “give it a go” and learn by doing. We would suggest that “pragmatic” decisions made to tailor co-production to specific project circumstances are transparently reported, acknowledging where compromises to ideal co-production are made, and why.

The included studies reported many challenges to co-production—for service users, researchers and organizations, and in relation to the practical “doing” of co-production. To resolve these challenges, one could argue that the publication of unsuccessful cases and reflective pieces that tease out lessons learned would be most helpful; but this is generally not an ambition of researchers or publishers, so these examples remain hidden. Failure drives learning and the greatest learning happens when things go wrong, and some of the studies seemed to support this view [29, 36, 52, 61, 87]. Perhaps new journals coming online such as *Research Involvement and Engagement*, which is co-produced by patients, academics, policy-makers and service users, will lead the way.

Perhaps the most important finding was the evident call for practitioners to share experiences of co-production in practice, to help others better operationalize the principles. There was demand for reports of co-produced research to elaborate context and help determine which co-production activities are best suited to which projects or which projects and interventions are likely to best suit co-production. Some authors argued for procedural steps or principles for co-production. However, we believe there may already be too much guidance and prescription, and instead the applied health research community needs practical and financial support to enact and operationalize co-production. On a practical level, co-production needs to match the context, actors and purpose of new projects, and researchers need to organize structural, personal and organizational factors to set up the right conditions from the start. Financial support is required to fund people with the skills to carry out co-production and time for people to accomplish co-production over the long term. Most current funding models support the practical conduct of research but fall short of investing in relationship- and network-building over time. A democratically driven vision of producing research with patients, the public and other stakeholders across projects and over time will only become a reality with a commitment to fund and support it.

### Limitations of the review

We intentionally included only papers reporting applied health research conducted in the United Kingdom—partly to keep the review manageable (the literature on co-production is extensive) and partly to keep the focus on learning within a specific context. A large proportion of the applied research conducted in the United Kingdom is funded by the NIHR, which directly reflects United Kingdom government and policy priorities; research groups often secure repeated funding for programmes, some of which have now spanned more than a decade. We felt it was timely to learn about co-production practice within these groups and identify priorities for future research funded within similar schemes and infrastructure. We acknowledge that the themes identified in the scoping review may not be generalizable to applied research conducted in other countries or under other funding arrangements. Our initial database searches included papers published from 2010 onwards, when “co-production” began to appear in the health literature and as a requirement of some funding schemes in the United Kingdom; we subsequently limited the date range to 2018–2020 due to the large number of hits and to keep the data extraction and synthesis manageable. We recognize that we may have missed important work that could contribute to our findings. However, this is a scoping review, conducted to rapidly map the recent literature, and not an exhaustive systematic review. We only had one author screening titles and abstracts, and independent screening of 25% of full texts due to researcher time and resource availability; we received no additional funding to conduct the review. We acknowledge that there is a small risk of selection bias through exclusion of eligible studies.

## Conclusion

This scoping review provides ample evidence that complex health interventions, service improvements and applied research are being co-designed and co-produced with patients, the public and other stakeholders, and supports current knowledge about the diverse processes and formats of co-production. However, what is clear from this review is that researchers are operationalizing co-production in various ways, often without the necessary financial and organizational support required and the right conditions for success.

Instead of trying to define a gold standard in co-production, we argue for accepting the diversity in approaches to co-production and call on researchers to be clearer in their reporting. Different approaches are needed to tailor co-production to context, different stakeholder groups and various stages of the research and implementation process. To assess which approaches are best suited in which context, for which groups and at what stage, researchers should be more reflective on the use of their chosen approaches in practice and be more systematic in reporting their learning (including failures) to allow for better operationalization of co-production principles and guard against tokenistic use of the term “co-production”.

As a minimum, researchers should record:

- a description of activities they undertake as part of co-production;
- which stakeholders were involved in this process and in what way ways, with a particular emphasis on how power is shared between stakeholders;
- the stages of the research and implementation process these stakeholders were involved in;
- skills that were developed by participants (including researchers); and
- the desired and achieved outcomes of these activities and the methods used to assess these outcomes.

Instead of being overly prescriptive about these different reporting elements, we argue that there is value in regarding co-production as an exploratory “social space” and a generative process, rather than a means to deliver impact and outputs, in order to encourage people to “give it a go” and learn by doing. However, without adequate resources and institutional support for people to work co-productively across projects and over time, the key principles become harder to enact, and innovation and creativity in collaboration and involvement in research is likely to be stifled. Entrenched academic and scientific practices are an apparent impediment to progress in co-producing applied health research. Changes are required at the individual researcher level, such as embracing more equal power-sharing, accepting different forms of evidence production, refining negotiation and communication skills and managing stakeholder relationships. Organizational changes such as proper governance and research policies that enable co-production are likely to take longer. As a result, the democratically driven vision for co-production—that it is the right thing to do and the endeavour in itself is sufficient to realize outcomes—which so many funders and commissioners of applied health research aspire to, and many researchers believe in, remains out of reach. In spite of these challenges, our scoping review has highlighted different ways of realizing co-production in applied health research, which can be further refined and researched within current research infrastructures such as the NIHR Applied Research Collaborations.

## Supplementary Information

**Additional file 1: **Example search strategy for MEDLINE (adapted for other databases).

**Additional file 2: **Conceptualization and implementation of co-production in the included papers.

## Data Availability

The datasets used and/or analysed during the current study are available from the corresponding author on reasonable request.

## Abbreviations

CLAHRC: Collaborations for Leadership in Applied Health Research and Care
EBCD: Experience-based co-design
INVOLVE: A national advisory group funded by the NIHR (no longer active). Taken over by the NIHR Centre for Engagement and Dissemination in April 2020
NIHR: National Institute for Health Research
PPI: Patient and public involvement
PPI/E: Patient and public involvement/and engagement
PRISMA-ScR: Preferred Reporting Items for Systematic reviews and Meta-Analyses extension for Scoping Reviews

## Footnotes

1 https://www.learningforinvolvement.org.uk/?opportunity=nihr-guidance-on-co-producing-a-research-project
