# Supplementary material for: Co-production practice and future research priorities in United Kingdom-funded applied health research: a scoping review
Source: Health Res Policy Syst. 2022 Apr 2;20:36. doi: 10.1186/s12961-022-00838-x (PMC8976994; doi:10.1186/s12961-022-00838-x)
Supplement: Supplementary file 1 — Additional file 1: Example search strategy for MEDLINE (adapted for other databases). [file 12961_2022_838_MOESM1_ESM.docx]

Additional file 1. Example search strategy for MEDLINE (adapted for other databases)

| **Search string** | **Content** |
| --- | --- |
| #1 | (co-production or co-design or co-creation or co-evaluation or co-produc* or co-creat* or co-evaluat*).m_titl |
| #2 | Limit 1 to yr=”2010-2020” |
| #3 | (applied health research or health research or healthcare research of health care research).m.titl |
| #4 | Limit 3 to yr=”2010-2020” |
| #5 | 2 and 4 |
