# Supplementary material for: Co-production practice and future research priorities in United Kingdom-funded applied health research: a scoping review
Source: Health Res Policy Syst. 2022 Apr 2;20:36. doi: 10.1186/s12961-022-00838-x (PMC8976994; doi:10.1186/s12961-022-00838-x)
Supplement: Supplementary file 2 — Additional file 2: Conceptualization and implementation of co-production in the included papers. [file 12961_2022_838_MOESM2_ESM.docx]

Additional file 2. Conceptualisation and implementation of co-production in the included papers

| **Paper** | **Term Used** | **Definition contained in the paper** |
| --- | --- | --- |
| Ali, 2018 | Co-design | Our co-design approach involved **active participation** by local people. Initial insight generation employed **six focus group discussions** and **14 individual interviews** to describe current understandings and information needs. A total of 11 personas (heuristic narrative portraits of community 'segments') resulted; **four participatory workshops** provided further understanding of: preferred information channels; feasible information conveyance; and responses to existing materials. Prototype information resources were then developed and **feedback gathered via two workshops**. Following further refinement, **final feedback** from health-care professionals and community members ensured accuracy and appropriateness. |
| Ball, 2019 | Active PPI | **Patient and Public Involvement** (PPI) in health research can take place across different stages of a research cycle, for example through: (i) contribution to priority-setting exercises or specification of research questions; (ii) developing or reviewing funding applications; (iii) helping design studies; (iv) assisting with recruitment of study participants; (iv) engaging with data collection and data analysis; (v) facilitating dissemination and research uptake; or (vi) contribution to evaluation activities.  This review is primarily concerned with **active PPI** in healthcare research. Active involvement entails, for example, **helping shape research questions or priorities**, or **contributing to data gathering, analysis or interpretation**. Considering patients and the public as study participants (e.g. participants in trials or interviewees for a study as part of the research methods) does not qualify as active involvement. |
| Barnett, 2020 | Co-production/ Co-creation | We consider the problem of **co-production of knowledge** about One Health and zoonotic diseases in relation to funding structures, working in large international teams and explore some of the often-neglected realities of working across disciplines and cultures.  **Co-creation of knowledge** which involves **shared decision-making** demands that we recognise co-production as a challenging process requiring time, role clarity, constant attention to power imbalances, difficult discussions about research rigour versus research relevance, and constant monitoring |
| Bench, 2018 | PPI | Explore the extent and nature of evidence on **service user involvement** in critical care research and quality improvement. Involvement is most commonly reported at the level of **consultation or participation in project teams**, however, the extent to which involvement impacts on projects output remains unclear.  **Patient and public involvement** (PPI) in healthcare is strongly endorsed by the National Institute for Health Research (NIHR) in England. The NIHR funded body that promotes public involvement in England (INVOLVE), defines PPI as **projects carried out ‘with’ or ‘by’ members of the public** rather than ‘to’, ‘about’ or ‘for’ them (Denegri, 2015). Within critical care, PPI usually refers to the **involvement of former patients and their family members**, who are described as service users. |
| Beal, 2019 | Co-production | Review the literature on how **co-production** is being used in mental health settings. We understand co-production to be: **A relationship where professionals and citizens share power to plan and deliver support together**, recognising that both partners have vital contributions to make in order to improve quality of life for people and communities. |
| Beresford, 2019 | Co-production/ PPI | Exploring the **Co-production of Knowledge. Public participation** in health and social care.  Here it is suggested that an essential first step to advancing **public participation in health** and the advancement and **co-production of knowledge** within it, is to put it in the context of developing modern democracy more generally. |
| Best, 2019 | Co-production | Promote the use of **co-production** in mental health practice. Innovative approaches to **sharing and developing co-production skills and attitudes** in mental health student nurses.  Common themes include setting the environment, developing a common aim and relationship building. |
| Bielinska, 2018 | Co-design | **Co-design** with patients carers and healthcare professionals can generate more detailed meaningful data through better conversations. |
| Bolton, 2020 | Co-production | The importance of **co-production and community control.**  We evaluated the project using two studies. In one, the sampling frame and methodology were **under community control**, permitting a comparison of change in outcomes before and after participation in the project. In the other, the sampling frame and methodology were under researcher control, permitting a case-control design. |
| Chisholm, 2018 | EBCD | **Experience-based** **co-design** (EBCD) is a service design strategy that facilitates **collaborative work between professional staff and service users** toward common goals. EBCD engages those who use services and staff at “**every stage within the design process, from problem diagnosis to solution generation and implementation**”.  The idea of “co” in co-design refers to moving service users to a position where they are **actively contributing to designing care**. |
| Connolly, 2020* | Co-production/ Co-creation | How **co-production** and **co-creation** is understood, implemented and sustained within the health and social care system.  Our analysis of the literature found that **co-production and co-creation are largely very similar** but **co-creation points to the sustainability of the role of the user throughout the process** of programme design, development, implementation and evaluation (not just at the programme development and design stages). |
| De Andrade, 2020 | Co-production | **Co-production** to improve health and address inequalities. Co-production involves **equal and active involvement of partners** including communities, health practitioners and voluntary sector **in the design and delivery of services**, programmes, or initiatives. |
| Dent, 2019 | Co-production | **Co-production** in mental health. This paper discusses one approach to **involving service users in planning**, namely, appreciative inquiry (AI).  Participants were organised into pairs, mostly with a service user and a staff member working together, to **interview staff and service users about their best experiences** of care planning and ideas for improved practice. The project was bookended by the hosting to two trust-wide workshops comprising service users, carers, trust staff and other stakeholder to first discuss their past experience of good care coordination and help generate ideas for change. |
| Dowie, 2018 | SDM/ Co-creation | **Shared-decision making** or **co-creation**. The coming paradigm will therefore be characterised by apomediative (‘direct-to-consumer’) decision support tools, engaged with by the person in the community to help them make health production decisions for themselves (including whether to consult a healthcare professional or provider), as well as intermediative (‘direct-from-clinician’) tools, delivered by a health professional in a ‘shared decision making’ or ‘co-creation of health’ process. |
| Eades, 2018 | Co-production | A **co-produced survey** measuring self-reported changes to patient self-determination. The questionnaire was **co-produced with a focus group of patient volunteers** resident in the hospital. |
| Farr, 2019 | Co-production/ Co-design | Implementation of **co-designed software for co-production** in mental health care planning  An electronic care pathway tool (CPT) was **co-designed** by service users, staff and software developers, to facilitate co-production of care and crisis plans. |
| Faulkner, 2021 | Co-production | An example of service user leadership and **co-production** in mental health research. **A user-led study** investigating mental health service user experiences of targeted violence and abuse. The research was both user-led and carried out **in collaboration with practitioners and academics**, a form of research co-production. |
| Fletcher, 2020 | Co-production/ PPI | The evidence supports the normative claim that health research regulation should continue to move away from strict, prescriptive rules-based approaches, and towards flexible principle-based regimes that allow r**esearchers, regulators and publics to co-produce** regulatory systems serving core principles.  **Co-production and collaboration** at different junctures of the health research endeavour emerged as important themes within the survey. Many of the Delphi participants responded to our questions about **public engagement** by discussing **public-patient-involvement (PPI) or public involvement (PI)** instead. |
| Gartshore, 2018 | EBCD | **Patient involvement in the design of health care** is a key policy initiative but few empirical studies address this. The main aims were to: (1) study the implementation of a service user-led **Experience-Based Co design**. |
| Gault, 2019 | Co-production | Aim: To **co-produce consensus on the key issues** important in educating mental health-care professionals to optimize mental health medication adherence in BAME groups. Using individual **interviews and a consensus workshop with carers and service users**.  For the purposes of this study, co‐production is defined as **a process in which people who use a service are involved in all stages of the development of a new service** or initiative. |
| Giebel, 2019 | Co-production/ PPI | This paper had two aims: (a) to assess the extent of public involvement and (b) to explore the experiences of public advisers in the dissemination of the HHS. This was framed around the NIHR INVOLVE guidelines on **public involvement.** **PPI** can take many shapes and forms, from identifying research priorities, to developing questionnaires and interpreting and disseminating findings.  A **public workshop** was set up to aid the **co-production** of the research evidence. This group fostered the co-production of research together with members of the public and aimed to adhere to the six principles of good public involvement as outlined by INVOLVE. |
| Girling, 2019 | EBCD | The aim of this research was to explore...(2) how **Experience-Based Co-Design** could be applied to facilitate recognition and service developments for young people with mental health difficulties. EBCD is an approach to healthcare improvement that enables **staff and service users to jointly co-design services.** |
| Green, 2020 | EBCD | **Experience-based codesign** is an approach to health service design that **engages patients and healthcare staff in partnership to develop and improve health services** or pathways of care. We defined the minimum requirements to be considered EBCD for this review as including **two phases where service users were participants in both phases**. |
| Green, 2019 | Co-production/ PPI | There has been a discernible shift in the narrative of PPI and a change in terminology. There is now a greater emphasis on patients and the public having a stronger voice in order to **share decision-making to co-produce research**, services, and policy.  A further distinct feature of this collection is the **contribution of service users both as authors** offering powerful user-led perspectives, **and as part of the editorial process.** |
| Halsall, 2019 | Co-production/ Co-design | The use of social media by creating a **Facebook forum for discussion and consultation**. A closed group of members with either lived or professional experience of perinatal mental health issues was **encouraged to interact through a structured forum to discuss developments**. T**heir knowledge, thoughts and suggestions were used to inform the project and their opinions sought about proposed design**.  In conclusion, the venture achieved **some elements of co-production, although co-design may be a better descriptor** of the final result. |
| Halvorsrud, 2019 | Co-creation | Effectiveness of **co-creation/production** in international health research. Alongside others who employ the term ‘co-creation’ to describe creative and collaborative activities to improve human experience, we privileged **‘co-creation’ as an umbrella term** for this particular review **to describe the involvement of non-academic stakeholders in research.** However, the terms ‘co-creation’, ‘co-production’ and ‘co-design’ are often used interchangeably although each relates to different processes, and all three descriptors were retained in the search strategy.  References were included if they sufficiently described non-academic stakeholders’ co-creation with researchers in the design and/or implementation of the research project |
| Hannigan, 2018 | Co-creation/ co-design | Ethnic Minority Health in Ireland—**Co-creating knowledge**.  A participatory health research study where all stakeholders, including ethnic minority communities, participate in **co-design of the research protocol, project governance, collaborative data interpretation and disseminating findings.** |
| Hoddinott, 2018 | Co-production/ PPI | Patient perspectives can be sought through **patient involvement** and through patients participating in **surveys, interviews or focus groups** to provide data for others to analyse, interpret and act on. Partnership research methods involve patients, clinicians, academics and other relevant stakeholders as **equal mutually respected partners in the research** team. Being a patient partner implies **equal opportunity and equal voice**. |
| Horgan, 2018 | Co-production | The COMMUNE project used a **co-production** approach, and experts by experience (people with experience of distress, service use, and recovery) were **partners in all stages of the research process, from grant application, through to dissemination**. |
| Horgan, 2020 | Co-production | This paper presents findings from a qualitative research project which were utilized to develop a framework entitled: Standards for **Co-production** of Education (Mental Health Nursing) (SCo-PE [MHN]). SCo-PE (MHN) includes **nine standards that were co-produced between nursing academics and EBEs** to support and assist in the design, delivery and evaluation of nursing curricula. |
| Hundt, 2019 | Co-production | This is a critical analysis of the **co-production of knowledge** on health care with **members of the public attending two research-based plays that were followed by post-show discussions** with expert panelists. The co-production of knowledge through the process and impact of research-based Theatre was experienced by both actors and audience members. This was further demonstrated in the way that audience members shared experiences during the participative post-show discussions.  The analysis provides critical reflections on the immediate, post-performance impact of research-based Theatre as a strategy to encourage the co-production of knowledge beyond delivery of the performance itself. The **plays were developed through partnership working from interview transcripts and joint workshops engaging academics, users and Theatre practitioners** (writers, director, actors). |
| Kaehne, 2018 | Co-production | The most ambitious versions of **co-production** preconceive of co-production as an activity taking place in a space governed by fairness, mutual recognition of interests, parity of access to resources, congruity of knowledge and familiarity with terminology... advocating **full accountability and full meaningful involvement of patients and users in the decision-making process.**  In contrast, a **pragmatic model of co-production** needs to articulate what the role and purpose of patients and users is within the process of service changes, drawing on realistic notions of patient capacity, and the user ability to influence complex and highly structured organisations. It recognises that what constitutes co-production depends to a large extent on our abilities to effectively navigate the interactions between professionals and users to produce an arguably co-produced service. |
| Kislov, 2019 | Co-production | **Co-production**, involving **collaboration with policymakers, clinicians and managers throughout the research process**. Co-production approaches differed depending on the stage(s) of the research process in which they were deployed as well as on the type of stakeholders involved. |
| Kislov, 2018 | Co-production | **Co-production** approaches, variously referred to as integrated knowledge translation, participatory research and co-design, provide an alternative to the traditional ‘push’ and ‘pull’ modes of translating research evidence into better healthcare. |
| Lambert, 2018 | Co-production | The aim of this discursive paper was to explore the development of **co-production** and service user involvement in UK university-based mental health research.  Co-productive approaches need to involve **service users collaborating with practitioner allies** to challenge and resist the restrictions of traditional, interpersonal, and structural power dynamics within research. For mental health research, this means more e**qual social relations of research production**, the empowerment of service users, and the making of broader social and political change. |
| Langley, 2018 | Co-creation/ Co-design/ Collective making | This discussion paper builds on this proposition and extends it beyond **knowledge co-creation to co-designing** evidenced based interventions and implementing them. Within a co-design model, we offer a specific **approach to share, mobilise and activate knowledge, that we have termed ‘collective making’.** It is this practice that has a direct influence on stakeholders themselves as co-creators in the process, creating the conditions for knowledge mobilisation.  Co-design has an **emphasis on process**, where facilitation **brings different participants together to elicit and share first-hand experiences** and first-hand knowledge perspectives. A move away from the opinion of ‘users’ as passive recipients of design work (designing for people) to **active participation in design processes**. |
| Leask, 2019 | Co-creation | More tailored solutions centred on individuals’ needs and circumstances can be developed in collaboration with these individuals. This process, known as **co-creation**.  Key principles and recommendations for using participatory methodologies in public health intervention co-creation are presented for the stages of: Planning (framing the aim of the study and identifying the appropriate sampling strategy); Conducting (defining the procedure, in addition to manifesting ownership); Evaluating (the process and the effectiveness) and Reporting (providing guidelines to report the findings). |
| Lignou, 2019 | Co-production | We argue for the value of including people with a diagnosis of mental health disorders and/ or their carers as co-researchers in mental health research. We claim that **co-production** shares key ethical values with the citizen science approach, such as the acknowledgment of the right of citizens to manage their own care and the **value of “expertise by experience**.  Co-production is a collaborative model of research implying an **equality of status** between stakeholders who contribute different skills and experiences. In co-production, sharing different kinds of knowledge and expertise emphasizes the value of mutual and continual learning among research partners. |
| Litchfield, 2018 | Co-design | **Two focus groups** convened to discuss suggestions for the improvement of blood testing and result communication in primary care. The groups were **mixed of patients and staff** in various combinations drawn from the four participating study practices.  By utilising the experience of patients, alongside those of a range of clinical and non-clinical staff to co-design service improvement interventions. A central principle of **co-design** is the provision of a platform where **all participants are able to express themselves openly**, question existing systems and methods of working, and explore alternative perspectives. In this way, interventions are developed born of the experience of all stakeholders and acknowledging their preferences and needs. |
| Lloyd-Williams, 2019 | Co-production | The WorkHORSE project was designed to **continuously engage with stakeholders to inform the development of an open access modelling tool** to enable commissioners to quantify the potential cost-effectiveness and equity of the NHS Health Check Programme. An objective of the project was to evaluate the involvement of stakeholders in **co-producing** the WorkHORSE computer modelling tool.  Iterative development of the decision support modelling tool was informed through **engaging with stakeholders during four workshops.** 30 stakeholders participated, of which 15 attended two or more workshops. |
| Luchenski, 2019 | Co-production | Using a **co-production** approach, we held a 1-day event in London, UK, that involved **inclusive, participatory, and consensus-building activities**. We facilitated **workshops** on preventing exclusion, improving services for excluded groups, and escaping exclusion. **We recorded participants’ views as observations, field notes, and ranked-lists of problems and suggested solutions.** |
| Marent, 2018 | Co-design | We initiated a **co-design** process with patients, clinicians and technology developers in five clinical sites. To implement co-design, we worked closely with clinicians and patient organisations. Together, we designed a protocol for engaging patients and clinicians alongside the iterative phases of the platform's design and implementation. It was decided to use co-design **workshops as main method to involve potential users in design activities to develop ideas and identify challenges.** To ensure flexibility in data collection, **individual interviews** were considered as an alternative where participants were unwilling or unable to attend workshops. |
| Metz, 2019 | Co-creation | To further explore the contribution of **co-creation** to support the use of evidence in policy and practice change. For this pursuit, we focused on co-creative capacity, which we defined as ‘**the deep involvement of a range of key stakeholders** across scientific, governance, and local practice boundaries **to create the infrastructure and context that enables and sustains the use of evidence in practice**’. While some of these have discussed ‘co-production’, the term ‘co-creation’ has also been used in the knowledge mobilisation literature to describe **close, collaborative working.** |
| Miles, 2018 | Co-production | Slow co-production, which entails **involving patients in qualitative, in-depth research from the start and throughout the project**. We have previously defined co-production as an “exploratory space that brings together different values and social relations”.  The project was **conceptualised and designed with the patients and patient representatives**. We collaboratively designed This Sickle Cell Life with people with sickle cell and their carers from the earliest proposal and planning stages. Our in-depth, qualitative research includes **repeated interviews and participant diaries** with young people with sickle cell, to facilitate prolonged, contextual, and more personalised engagements with participants. We are working with them and with other stakeholders (patient charities, clinicians, service improvement experts) to **coproduce support resources** based on our study findings. |
| Norton, 2019 | Co-production | **Co-production** is a word used to describe the creation of a dialogical space where the service user, family members, carers and service providers enter **a collaborative medical partnership** to improve their own care and also service provision. |
| O'Connor, 2019 | Co-design | **Co-designing a mobile application** with people with dementia and their carers. In-depth **interviews** with people with dementia, their carers, and others involved in co-creating a mobile health application were conducted. |
| Pallesen, 2019 | Co-design | **Co-design involves stakeholders as design partners** to ensure a better fit to user needs. Co-design approaches use the real-life experience of patients and health-care providers to improve service design and delivery. It goes beyond user involvement, where end-users have a consultant or advisory role. In co-design, stakeholders are involved as **equal partners and co-creators**.  Semi-structured **interviews** were conducted with individuals (n = 10) who took part in the **co-design workshops**. Twenty-one people took part in the co-design process (7 researchers, 12 HCPs and a patient representative). A seventh workshop was held with five patient  representatives and advocates to ensure additional PPI input. |
| Palumbo, 2018 | Co-production | The **co-production** idea assumes that the synergies between the users and the providers in the **design and delivery of public services** pave the way for the establishment of a co-creating partnership, which is able to enhance the process of public value creation. |
| Patel, 2018 | Co-production | Three London care homes **co-produced a pilot oral health training programme**, informed by in-depth **interviews and group discussions.** Having identified the key issues, a training programme was developed with mock-ups of resources, aligned with the national messages, and shared in draft with the care home teams, who commented on the structure and logistics of delivery. |
| Pearce, 2020 | Co-creation | We have achieved our aim of defining **co-creation of new knowledge** as:  The generation of new knowledge that is derived from the application of rigorous research methods that are embedded into the delivery of a program or policy (by researchers and a range of actors including service providers, service users, community organisations and policymakers) through **four collaborative processes**: (1) generating an idea (**co-ideation)**; (2) designing the program or policy and the research methods (**co-design**); (3) implementing the program or policy according to the agreed research methods (**co-implementation**), and (4) the collection, analysis and interpretation of data (**co-evaluation**). |
| Ponsford, 2020 | Co-production | **Co-production** of two whole-school sexual health interventions for English secondary schools. The initial design of both interventions was primarily informed by academic theory and research. However, the drafting of the funding proposals for each study also involved **preliminary consultation with a staff member** from five different schools and with young people from a young people’s research advisory group.  Formative **qualitative inquiry** involving 75 students and 23 school staff. Detailed written summaries of **workshops and events** were prepared and **key themes identified to inform the design of each intervention**. |
| Realpe, 2018 | Co-production | **Co-production** refers to the **contribution of service users to the provision of services**. Collaborative co-production challenges the usual relationship between professionals and service users. It requires the latter to be considered experts in their own circumstances and therefore capable of making decisions and having control as responsible citizens. At the same time, co-production also implies a change in the role of the professionals from fixers of problems to facilitators who **find solutions by working with their clients**. |
| Rodriguez, 2019 | Co-design | The aim of this qualitative exploration was to use critical consciousness as an educative tool, to **co-design**, implement, and evaluate a series of oral health and health pedagogical workshops.  13 young people and five staff members from this NGO **participated and co-designed eight workshops**. A series of meetings took place between the PI and managers, staff members of the NGO, and young homeless people living in temporary accommodation, with the intention of co-designing the content and delivery of the workshops. |
| Rose, 2019 | Co-production | This paper examines the concept and practice of **coproduction** in mental health. “a **relationship where professionals and citizens share power**” and recognize the contributions that each actor makes to this process. |
| Scott, 2020 | Co-design | Families attended **two co-design workshops** at a Family Hub in Portsmouth, during which they discussed oral health priorities and commented on the animation design. A before-and-after survey questionnaire assessed its impact on oral health knowledge in another Family Hub in Portsmouth. |
| Sherriff, 2019 | Co-production | **Co-producing knowledge** of LGBTI health-care inequalities. Rapid **reviews of grey literature were co-produced with LGBTI people** in 27 countries followed by a thematic analysis and synthesis across all data sets. These LGBTI contacts were **involved in every stage from designing the template, identifying the literature and summarizing content for their country.**  The principles underpinning PPIE include **actively involving service users in research** and the organizations that conduct research, and involving service users in sharing knowledge of the research with the public. |
| Slattery, 2020  * | Co-design | The aim of this study was to provide an accessible synopsis of current **co-design** approaches and activities in health research.  The definitions used to scope this review were informed by the PCORI. We defined ‘research co-design’ as the **meaningful involvement of research users during the study planning phase of a research project**, where ‘meaningful involvement’ is taken to refer to participation in an explicitly described, defined and auditable role or task necessary to the planning and/or conduct of health research. |
| Smith, 2020 | Co-design | Stakeholder **engagement and co-design in healthcare**. The qualitative nature of patient experience must be recognised and **given equal priority to that of healthcare professionals**. It is therefore important to i**nvolve patients, clinicians, and managers early in the improvement initiative** and select methods that allow them to work together on improvement.  This includes facilitating conversations between stakeholders about what matters to them and **creating opportunities for practical and tangible improvement activities** such as small-scale tests of change, working through the plan-do-study-act cycle, or creating prototypes together. |
| Syed, 2019 | Co-creation | **Co-creation of evidence** is therefore defined as any approach **to integrate best available research evidence together with best available other types of evidence such as patients’ expectations**, clinicians’ perspectives, local data, resources and so on to inform EIPH/EIDM. This is consistent with the definition of co-creation in the published literature |
| Tembo, 2019 | Co-production/ PPI | How the public can be involved in the **co-production of research commissioning** early on in the process. The organization “consumers in research” now known as INVOLVE, a national advisory group for PPI. A legacy of this organization is its widely used definition of PPI, which we utilize in this paper: “**Research being carried out “with” or “by” members of the public rather than “to”, “about” or “for” them**”.  Co-produced research harnesses the **principles of sharing of power**, including all perspectives and skills, respecting values and the knowledge of all those working together on the research, reciprocity and building and maintaining relationships. |
| Thompson, 2020 | Co-production/ Co-governance | **Co-production and co-governance** in the Scottish National Health Service. Simply put, co-production describes a **voluntary relationship between citizens**, as service users or members of voluntary associations, **service providers and governmental organisations, whereby public services are produced and delivered in partnership.**  One fundamental distinction is between individual co-production and collective organisational co-management and co-governance. Co-production can involve one or more elements of the production process, from design through implementation to evaluation and recommendations, which are considered here in respect of the core tasks of the service. Co-governance engages civil society organisations in offering advice, support and material contributions to health and social care. |
| Tribe, 2019 | Co-production | This paper will discuss examples of **mental health training developed and co-produced in active partnership with two communities**. In community partnerships, cultural understandings of psychological distress can be shared and community partnerships developed which can lead to the **co-production of resources and mutual learning**. This could be viewed as a form of **bidirectional training of both community workers and western trained clinicians**.  After a series of meetings, the author and two colleagues (**a psychiatrist and two psychologists) and the community workers co-produced a program of 12 training sessions** which took account of local, cultural and situational factors. Volunteer community workers from the community center attended, along with a number of clinicians/trainers who hoped to gain knowledge and understanding of cultural and refugee issues from the volunteers. |
| Whitham, 2019 | Co-design | The **co-design** activity takes a novel approach, explicitly engaging health and social care staff and service users in the co-design of tools for use in their future work. We present three case studies of tool co-design projects. We also take a critical perspective on our co-design approach.  Researchers worked with individuals who had direct, lived experience of health and social care services, unpacking key challenges service professionals and services users faced, then developing tools they might use to structure and restructure conversations. Following a Participatory Action Research approach, for each initiative the research process consisted of (1) **interventional tool co-design activities** with 5-30 participants over multiple events, (2) **tool-sharing activities** to disseminate co-designed tools across practitioner communities, (3) **evaluation activities** looking for long term change in the partner organisations, and (4) ongoing evaluation activities examining the practice of tool users over time. |
| Wolstenholme, 2019 | Co-creation/ Co-production | ‘Mode 2’ **recognises different forms of knowledge**: practical knowledge, tacit knowledge, procedural knowledge and local knowledge, and recognises that this knowledge has to be generated within and be sensitive the context of its use. This process of ‘doing’ mode 2 knowledge mobilisation has been described as **co-creation or co-production**.  Co-production... in its best form speaks to a **genuine and meaningful engagement** between the key stakeholders to generate new knowledge that is sensitive to context and thus is more likely to be implemented.  First, we met to discuss current stroke service provision and to develop a map of available weight management stroke services. Second, co-production took place. **Two workshops** were used to collaboratively identify areas for development and to generate ideas for how changes could be made in service delivery and resource provision to addresses key gaps. |
| Brookes, 2019 | Co-design | This is a 3 year mixed-methods observational study using patient and staff **co-design** techniques.  Local project teams **including staff and patient**s from the acute medical units (AMUs) and intensive care units (ICUs) of three National Health Service trusts will implement **two experience surveys** derived from existing instruments: a continuous patient and relative survey and an annual staff survey. Outputs will be collated by the central project team to develop a reflective learning framework and toolkit which will be **fed back to the local groups for review, refinement and piloting**. |
| Buckley, 2019 | Co-production | 32 adults with controlled lifestyle-related health conditions took part in a physical activity referral intervention (**co-produced by a multidisciplinary stakeholder group**). We co-produced a PA referral intervention with commissioners, practitioners, service-users and academics. |
| Buckley, 2018 | Co-development | An ERS was iteratively **co-developed** by a multidisciplinary stakeholder group (commissioners, managers, practitioners, patients and academics) via **five participatory meetings and an online survey**. |
| Clayson, 2018 | Co-production | Reflexive data collection and analysis on processes of **co-production research**. It involved community organisation to facilitate data collection via participant-controlled computer-assisted structured interviewing (The VoiceBox), using a **co-production approach to method design, data collection and interpretation**. Involved partners were academic researchers, in-recovery community members and a community intermediary. A key aspect of this series was **the incorporation of people in recovery as VoiceBox, “crew” as community researchers; design consultants, recruiters/outreach, interviewers and data interpreters**. |
| Davies, 2019 | Co-production | **Designing and developing a co-produced theoretical and evidence-based online support** for family caregivers of people with dementia at the end of life.  Adopting an iterative approach and co-production methods the development process consisted of four stages: Stage1-Synthesis of data: three sources of data (interviews, systematic review and theory) were synthesised using tabulation, to identify the targets of the prototype; Stage2-Identifying intervention targets and components: a research development group (health practitioners, **a family caregiver** and academic experts) met to discuss the development, using a modified nominal group process, refining the synthesis from stage 1; Stage3-Developing the intervention prototype: an outline of the prototype was developed based on stage 1 and 2; and Stage4–User testing: interviews with caregivers testing the prototype website. |
| Evans, 2019 | Co-production | We recruited people with experience of chronic conditions, as patients and carers, and supported them to develop and implement the involvement model. We collected written records to describe the processes of **co-production**.  We held **one workshop** for all interested service users to enable them to develop the involvement model from the start. Through group discussion, they identified principles which they agreed should underpin a model for collaborative involvement in research. |
| Farr, 2018 | Co-production | To examine patient and staff views, experiences and acceptability of a UK primary care online consultation system and ask how the system and its implementation may be improved. Qualitative interviews with 23 practice staff in six practices. Patient survey data for 756 e-consultations from 36 practices, with free-text survey comments from 512 patients, were analysed thematically.  **Service co-production** can be understood as a process where **service quality is shaped by (1) people’s initial expectations of a service, (2) staff and service users’ roles, interactions and experiences within a service**, leading to (3) their resulting satisfaction or dissatisfaction. |
| Gradinger, 2018 | Co-production | Researcher-in-Residence’ (RiR) model which seeks to **co-produce knowledge** for action. It suggests researchers should: 1) be co-located with or embedded in the services for significant amounts of time and have a stake in the research or improvement initiative they are supporting; and 2) negotiate the meaning and use of research-based knowledge and co-produced knowledge that is sensitive to the local context and responsive to the continuously changing needs of the system. |
| Henshall, 2018 | Co-production | **Co-production and evaluation of an intervention package**. The study design comprised of three stages: (1) The first stage included **focus groups** with midwives to explore the barriers to carrying out place of birth discussions with women. (2) In the second stage, COM-B theory provided a structure for **co-produced intervention development** with midwives and women representatives; priority areas for change were agreed and the components of an intervention package to standardise the quality of these discussions were decided. (3) The third stage of the study adopted a mixed methods approach including **questionnaires, focus groups and interviews with midwives to evaluate the implementation of the co-produced package in practice.** |
| Hubbard, 2020 | Co-production | **Co-production** of “nature walks for wellbeing”. A multidisciplinary team of seven academics and health practitioners, together with service user input, developed an intervention in 2018 by **scoping the literature, face-to-face meetings, email and telephone**.  Five two-hour meetings were held between members of the research team, charity representatives and health practitioners. The purpose of the meetings was to draw on practical knowledge and expertise to inform intervention development. Hence, the meetings were designed to bring two sets of expertise – those who use research evidence and theory drawn from the literature and those who use practice know-how to develop interventions - together to make key decisions. |
| Lim, 2020 | Co-production | Evaluating hospital tools and services that were **co-produced** with patients. Search terms from these concepts were included: (i) co-production (e.g. **co-production, co-design, co-production, coproduction, co-creation and cocreation)**. The inclusion criteria were studies that... (iii) **included patients in health service improvement activities**. |
| Locock, 2019 | Co-production/ Co-design/ PPI | This paper examines different **approaches to involving patients and family members in biomedical research**, particularly in designing interventions to be tested in randomised controlled trials. |
| Madden, 2020 | Co-production/ PPI | A 2015 strategic review of NIHR PPI suggested **co-production, ‘as a means of evolving and improving public involvement in research**,’ recommending the adoption of co-production principles to foster partnership, reciprocity and openness.  The CHAMP-1 programme is co-producing with the pharmacy profession and with patients and carers, a complex intervention for discussion of alcohol within community pharmacy-based medication reviews. **Patient involvement was initiated before drafting of the CHAMP-1 funding application commenced** in a preliminary process.  The role of the PPI group is to **provide a patient perspective throughout the life of the programme** by advising the research team on the content and conduct of the research. Outcomes of this are recorded in a PPI impact log. The group met four times in the intervention development phase of the programme and is scheduled to meet twice a year thereafter. |
| O'Caithan, 2019 | Co-production | Partnership approaches included **co-production with equal participation in decision-making of the research team and the people whom the intervention aimed to help**, and user-driven development. |
| Ramaswarmy, 2020 | Co-production methods? | This chapter provides a framework and guidance on how clinicians and health-care leaders can work together to design, implement, and learn about ERAS improvement interventions more effectively |
| Raynor, 2019 | EBCD | **Experience-based co-design** (EBCD) brings patients and staff together to co-design services. The primary aim was to assess the feasibility and acceptability of conducting research-initiated EBCD.  We recruited four sites and, on each site, conducted the initial **traditional EBCD meetings**, with **separate staff and patient groups**—followed by **a single joint patient-staff event**, where four priority areas for co-design were agreed. Each site worked on one priority area, and the four co-design groups met over 2-3 months to design prototype tools. **A second joint event** was held where they shared and compared outputs. The research team combined elements of these outputs to create an intervention, now being tested in a cluster randomized controlled trial. |
| Rousseau, 2019 | Co-production/ Co-design | More widely adopted within health intervention development are the approaches of **co-production or co-design**; **the active involvement of key stakeholder**s–usually including the intended target population (in health terms this is often patients and the public), and those delivering the intervention. As with design thinking and human centred design, there are various approaches to co-design but all **share a commitment to power sharing** **in relation to design decisions** with these stakeholders. However co-design in particular, unless explicitly articulated, is a broad concept which varies in the level of involvement of stakeholders. |
| Smith, 2018 | Co-creation/ Co-production | **Co-production and co-creatio**n can be distinguished from ‘participation’, which, means being consulted and could also refer to passive involvement. In co-production **people who use services take over some of the work** done by practitioners. In co-creation, **people who use services work with professionals to design, create and deliver services.** |
| Young, 2019 | Co-production | We describe a process for **co-producing progression criteria** for an ongoing feasibility study. **Patient contributors, clinicians and researchers participated in discussions** facilitated using the modified Nominal Group Technique (NGT).  Progression criteria were developed using the NGT, which is a structured, systematic, transparent and inclusive method for both flexibly generating ideas, and quickly coming to consensus in a face-to-face environment. NGT involves: asking the group members to silently come up with ideas related to the given topic of discussion; sharing them; carrying out a group discussion; and finally voting and ranking them. These components **help facilitate the equal participation of all group members, promoting the sharing of power** and the ability for all to take a key role. |
